# Anatomy-Guided Hybrid CNN–ViT Model with Neuro-Symbolic Reasoning for Early Diagnosis of Thoracic Diseases Multilabel

**DOI:** 10.3390/diagnostics16010159

**Published:** 2026-01-04

**Authors:** Naif Almughamisi, Gibrael Abosamra, Adnan Albar, Mostafa Saleh

**Affiliations:** Department of Information Systems, Faculty of Computing and Information Technology, King Abdulaziz University, Jeddah 21589, Saudi Arabia; gabosamra@kau.edu.sa (G.A.); ambar@kau.edu.sa (A.A.); msherbini@kau.edu.sa (M.S.)

**Keywords:** hybrid CNN–ViT, anatomy-guided learning, Grad-CAM, neuro-symbolic reasoning, chest X-ray, thoracic disease classification

## Abstract

**Background/Objectives**: The clinical adoption of AI in radiology requires models that balance high accuracy with interpretable, anatomically plausible reasoning. This study presents an integrated diagnostic framework that addresses this need by unifying a hybrid deep-learning architecture with explicit anatomical guidance and neuro-symbolic inference. **Methods**: The proposed system employs a dual-path model: an enhanced EfficientNetV2 backbone extracts hierarchical local features, whereas a refined Vision Transformer captures global contextual dependencies across the thoracic cavity. These representations are fused and critically disciplined through auxiliary segmentation supervision using CheXmask. This anchors the learned features to lung and cardiac anatomy, reducing reliance on spurious artifacts. This anatomical basis is fundamental to the interpretability pipeline. It confines Gradient-weighted Class Activation Mapping (Grad-CAM) visual explanations to clinically valid regions. Then, a novel neuro-symbolic reasoning layer is introduced. Using a fuzzy logic engine and radiological ontology, this module translates anatomically aligned neural activations into structured, human-readable diagnostic statements that explicitly articulate the model’s clinical rationale. **Results**: Evaluated on the NIH ChestX-ray14 dataset, the framework achieved a macro-AUROC of 0.9056 and a macro-accuracy of 93.9% across 14 pathologies, with outstanding performance on emphysema (0.9694), hernia (0.9711), and cardiomegaly (0.9589). The model’s generalizability was confirmed through external validation on the CheXpert dataset, yielding a macro-AUROC of 0.85. **Conclusions**: This study demonstrates a cohesive path toward clinically transparent and trustworthy AI by seamlessly integrating data-driven learning with anatomical knowledge and symbolic reasoning.

## 1. Introduction

Pulmonary diseases continue to be a major global health challenge [1]. Globally, pulmonary diseases affect hundreds of millions of people and contribute significantly to the overall burden of morbidity and mortality. Timely and accurate diagnosis of pulmonary diseases provides effective treatment [2]. In addition, the early detection of some pulmonary diseases will help isolate the patient and prevent the spread of the disease. Chest radiography (CXR) is considered the main method for checking patients with pulmonary diseases because of its availability, low cost, and relatively low level of radiation [3]. Manual diagnosis is prone to errors because chest X-rays shown similar symptoms, making it difficult for doctors and specialists to differentiate between various lung diseases. This is compounded by differing opinions among physicians and a shortage of highly experienced specialists in this field. Therefore, artificial intelligence techniques can address the limitations of manual diagnosis [4]. Recently, Deep learning approaches have been applied extensively in the medical field, such as in the classification of medical images. CNNs have achieved strong performance in pathology analysis by extracting fine-grained spatial information and composing it into multilevel representations [5]. However, these models are not well suited to capture extended dependencies and holistic contexts across images [6]. This limitation is particularly significant for pulmonary disease diagnosis, where abnormalities frequently extend across regions and require whole-image context [7]. Vision Transformers have marked a considerable technique in computer vision, which has demonstrated state-of-the-art performance across various image classification tasks and has been adopted in various methods in medical contexts, including tasks such as identifying diseases in chest X-rays, although they require extensive computational resources and large datasets for effective training [8]. This has motivated the development of hybrid CNN-ViT architectures that combine the local feature extraction capabilities of CNNs with the global modeling power of transformers, offering superior performance for medical imaging applications [9]. However, most available deep learning solutions act as black-box models without explaining how decisions are made or providing any interpretation, which poses significant challenges for clinical adoption [10,11]. Therefore, the fundamental challenge in adopting AI models in the healthcare sector should be interpretability, trustworthiness, and robustness [11]. Many healthcare professionals remain cautious about relying on AI systems when the decision-making process is not verified or is unclear [12].

One of the most common problems, even with manual CXR interpretation, is misinterpretation due to the similarity of pulmonary diseases on CXR. This request AI system not only delivers high accuracy but also needs to be interpretable and transparent. The explainable artificial intelligence (XAI) method will help physicians understand the reasons behind a given disease’s prediction and classification, thereby building trust in the model [13]. Although recent studies have introduced several uncertainty estimation techniques tailored for medical AI, their thorough assessment in real-world clinical settings remains limited.

In medical imaging, ontologies provide a structured framework for knowledge and define how anatomical structures, imaging findings, and disease concepts are related to one another [14]. In practice, resources such as RadLex standardize the vocabulary and explicitly model the relationships between anatomical structures, imaging findings, and diagnostic entities [15]. By defining consistent vocabularies and logical links, ontologies ensure that annotations are interoperable, enabling powerful semantic searches and supporting automated reasoning, such as inferring likely diagnoses from a set of observed signs. Integrating these ontologies with deep learning models enables AI systems to combine data-driven predictions with knowledge-driven reasoning [16]. This structured layer makes ontologies a natural partner for Neuro-Symbolic AI, where neural networks contribute powerful feature extraction from complex images, while symbolic layers grounded in ontologies provide logical interpretability, clinical validation, and rule-based inference [17]. Together, these factors make the model more auditable and reliable.

The proposed study aims to create a clinically transparent AI system to classify thoracic diseases and incorporate explainable predictions and diagnostic reasoning into the model. The goals of this study were as follows: to build a hybrid EfficientNetV2-ViT architecture that enables robust feature learning; to build an anatomy-guided representation learning CheXmask; to build a neuro-symbolic reasoning layer that provides ontology-based clinical interpretation; and to build an extensive evaluation framework that guarantees both diagnostic accuracy and clinical verifiability across datasets and pathological conditions. This methodology combines the Enhanced EfficientNetV2, which reduces spatial features, with a modified ViT. CheXmask provides supervision for anatomical training and enables spatially aligned Grad-CAM visualization. A neuro-symbolic reasoning layer then processes these activations to produce clinically interpretable reports based on ontology-based diagnostic predicates using fuzzy-logic inference.

The main contributions of this study are as follows.

- A novel hybrid architecture that integrates an EfficientNetV2 backbone with a mixed channel–spatial attention mechanism, optimizing it for both fine-grained feature extraction in chest radiographs and computationally efficient, interpretable attention mapping.
- Anatomy-guided learning and interpretation through the integration of CheXMask segmentation masks. This provides auxiliary supervision during training to align features with lung and heart structures and later constrains Grad-CAM visualizations and symbolic reasoning to anatomically valid regions.
- The effective handling of severe class imbalance via a class-balanced focal loss improves the model’s sensitivity to rare thoracic pathologies without degrading its performance on common findings.
- A neuro-symbolic reasoning framework that employs a Logical Neural Network and fuzzy logic to translate anatomically grounded model activations into ontology-based diagnostic predicates. This moves beyond the probabilistic output to generate structured clinical rationales.

The remainder of this paper is organized as follows. Section 2 provides a literature review of prior work, emphasizing the methodological approaches and key findings of relevant literature. Section 3 describes the materials, dataset properties, and methodology applied to design and test the proposed system using the NIH ChestX-ray14 dataset. Part 4 presents the experimental findings on the NIH dataset and an independent external dataset, along with a detailed interpretation that includes neuro-symbolic diagnostic reasoning and ontology-based clinical explanation. Section 5 provides a comparative discourse that places the performance of the proposed system in the context of existing studies and highlights the system’s scientific and clinical contributions. Finally, Section 6 summarizes the main conclusions of this paper.

## 2. Related Work

The field of automated classification of pulmonary diseases is actively evolving as researchers seek more robust and generalizable models. The hybrid technique, which combines CNNs and transformers, has shown promising results; however, it still has limitations in terms of computational efficiency, interpretability, and reasoning.

Yanar et al. [18] examined several deep learning architectures, including CNN, Transformer, and Mamba-based models, for classifying chest radiographs using the NIH ChestX-ray14 dataset. All models were trained in the same manner to ensure the comparability of the results. Their results indicated that the hybrid architectures, especially ConvFormer, EfficientNet, and CaFormer, provided better diagnostic accuracy. ConvFormer achieved the highest mean AUROC score of 0.841. Baltruschat et al. [19] present a systematic evaluation of different approaches for CNNs to classify multi-label diseases from the ChestX-ray 14 dataset. They tested different loss functions and data augmentations for various ResNets. They demonstrated that a trained ResNet-50 from scratch achieved the best overall performance, with an average AUROC of 0.822 across all 14 classes. Jain et al. [20] examined the diagnostic performance of ViT for multi-label chest disease detection using the NIH Chest X-ray dataset. Two ViT variants, ImageNet-pretrained and from-scratch, were compared with CNN and ResNet baselines under identical experimental settings. Quantitative evaluation using multi-label accuracy and AUROC showed that the pretrained ViT achieved superior discriminative power and faster convergence; however, the analysis lacked clinical imaging analytics. Kufel et al. [21] presented a framework for classifying pulmonary disease using an EfficientNet backbone for feature extraction, followed by sequential layers including Global Average Pooling, Dense, and Batch Normalization. Binary cross-entropy loss was used to address multi-label classification in a single chest radiograph, achieving an AUC-ROC of 84.28%. Goel et al. [22] presented a Low-Rank Feature Learning (LRFL) framework to enhance thoracic disease classification on chest radiographs by suppressing noise and emphasizing diagnostically relevant regions. When applied to MAE-pretrained CNN and ViT models, the LRFL constraints learned representations to low-rank subspaces, yielding improved multiclass AUROC and accuracy compared to conventional methods. This approach effectively strengthens feature discrimination and generalization. However, its dependence on low-frequency assumptions may overlook subtle pathological variations in the high-frequency image regions. The practical clinical impact of this method, compared with other advanced regularization techniques, remains unquantified. Huang et al. [23] present a framework that can read and understand both medical imaging and radiology reports simultaneously. They developed a global representation learning framework (GLoRIA), which extracts representations using image and text encoders and learns global and localized representations. The used ReeNet-50 as a backbone with a transformer-based text encoder BERT using the MIMIC-CXR dataset. The model achieved an AUROC of 87% using only 10% of the labeled data. Chen et al. [24] optimized CoAtNet- using a loss function to address the long-tail problem in public datasets and multi-label classification. As a baseline and for comparison, they used a pretrained ResNet50 network with weighted binary cross-entropy loss (LWBCE). The baseline model, ResNet50 + LWBCE, achieved an AUROC of 0.811, whereas the model achieved 0.842. Group-CAM heat maps were shown for some categories, such as atelectasis, edema, effusion, and poor attention, but not for other categories, such as pneumothorax. Hanif et al. [25] used the pre-trained DenseNet-121 to classify lung disease from the NIH Chest X-ray 14 dataset, which is addressed below, and employed the rank-based loss, derived from Zero-bounded Log-sum-exp and Pairwise Rank-based loss, to address multi-label disease and class imbalance in the training data. The model achieved an average AUROC of 0.809. Chehade et al. [26] focused on image enhancement and correction; the images are cleaned of foreign objects such as medical devices, wires, and electrodes. They affect the model by making it difficult to differentiate relevant anatomical features, potentially rendering accurate diagnosis impossible. K-means clustering was used to separate the targeted image from the medical device, and without artifacts, the resulting image was then trained using CycleGAN to convert the dirty image to a clean one. The model achieved an AUROC of 84.96%. Additionally, the integration of multimodal data, as demonstrated by Tang et al. [27], has shown that combining chest radiographs with clinical data can enhance the prediction accuracy for conditions such as osteoporosis, further validating the utility of the NIH dataset for developing robust AI models. DSouza et al. [28] introduced a pre-trained ResNet-34 trained on ChestX-Ray14, fine-tuned using stochastic gradient descent with momentum, with restarts, and Variable image sizes to ensure no overfitting. An AUROC of 84% was achieved. Class imbalance was addressed using the noted baselines by computing the weighted binary cross-entropy loss and an asymmetric loss. The model was optimized using an Ensemble of Fine-Tuned DenseNet and Swin Transformer models, achieving an AUROC of 83.76%. Souid et al. [29] applied a pretrained MobileNet V2, fine-tuned together, to a classy fourteen-class dataset for NIH ChestX-Ray. The model produced an average AUROC of 81% DOI, and the DualCheXNet model, a two-fold asymmetric feature-extraction network, improved multi-class pulmonary disease classification in CXRs. This approach supports two different data-level fusions to enhance the complementary feature learning of DualCheXNet and achieves an AUROC of 82.3%. Chen et al. [30] presented two symmetrical CNN chains, as both models achieved success. One used ResNet-50 with Densenet-121, and the other used ResNet-101 and Densenet-169, with Support for two methods: data-level fusion and feature-level fusion. The model produced an AUROC of 82%. Ho et al. [31] enhanced the pre-trained DenseNet-121 using feature integration: Generalized Search Tree, Scale-Invariant Feature Transform, Histograms of Oriented Gradients, and Local Binary Patterns. The refined model generated an average ACU of 80.7%. Albahli et al. [32] presented a model framework, InceptionResNetV2, that enhanced the presentation of whole models. The model was used to classify 14 infection classes of the NIH dataset using pulmonary disease data features and achieved an AUROC of 80%.

Although previous studies have made significant progress in classifying thoracic diseases using CNNs, ViTs, hybrid models, and feature-regularization methods, several limitations remain. Studies lack anatomical supervision, leading to poor localizations and low clinical interpretability. Severe class imbalance, noisy NIH14 labels, and the absence of reasoning also affect several models. Additionally, the disconnect between Grad-CAM usage in previous studies and anatomical contextualization has reduced the reliability of the diagnosis. To make clinically meaningful and interpretable predictions, this study integrates anatomical supervision, a hybrid CNN-Transformer, class-balanced optimization, and neuro-symbolic reasoning.

## 3. Methods and Materials

**Figure 1:**
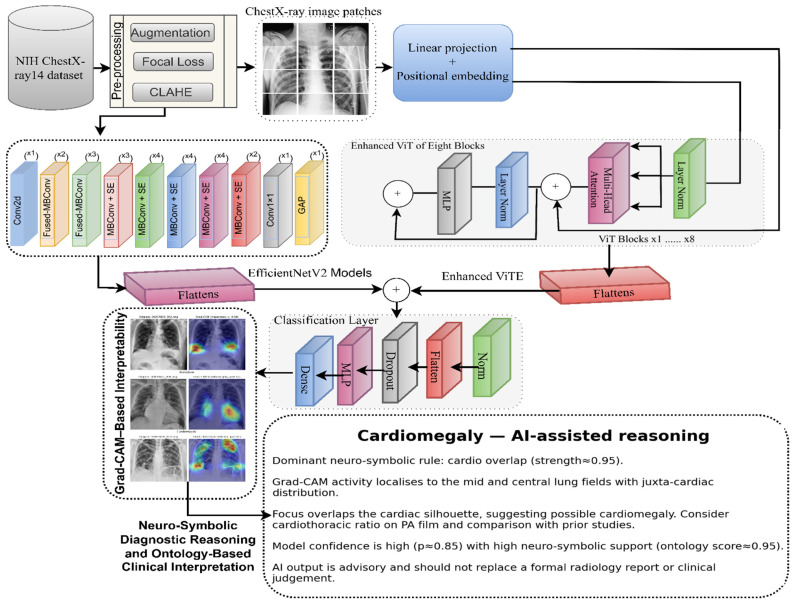
Proposed hybrid CNN–ViT model with integrated Grad-CAM and neuro-symbolic reasoning for thoracic disease classification.

The proposed methodology implements a hybrid CNN–ViT framework that combines local spatial representations and global contextual dependencies for the classification of thoracic diseases, beginning with standard preprocessing as Figure 1. The augmented images were then passed in parallel to the two branches.

The first branch architecture was the EfficientNetV2 model. This architectural pathway can extract multiscale local features and textures from pulmonary image data. The second branch, in parallel, implements a linear projection with positional embeddings, followed by a stack of eight transformer encoder blocks with multi-head attention, layer normalization, and MLP units to model the long-range spatial relations in the radiograph. The second branch of the architecture was designed to capture the global range relations in the image.

The outputs of all the branches were then flattened and concatenated to form a single representation. This single representation is processed by a classification head comprising dense layers, dropout, normalization, and an MLP block to obtain the final multi-label disease probability.

The Grad-CAM module in the model explains the model’s decision by generating a class-specific activation map that pinpoints diagnostic locations in both lung fields via a convolutional path. These visual explanations are paired with a lightweight neuro-symbolic reasoning layer that interprets image-based evidence using predefined ontological rules to explain predictions such as cardiomegaly or focal opacities.

Table 1 lists the components of the proposed system and their contributions.

### 3.1. NIH Chest X-Ray Dataset

The NIH ChestX-ray14 collection, made accessible by the National Library of Medicine and the NIH Clinical Center in 2017, has become one of the largest publicly available labelled datasets for medical imaging research. This study advances research on computer-aided diagnostic systems and the implementation of deep learning for the identification and localization of thoracic diseases. The collection features 81,176 frontal chest radiographs from 30,805 unique patients. It encompasses 14 thoracic diseases. The images were extracted from routine clinical examinations conducted at the NIH Clinical Center, providing a real-world continuum of variability in patient positioning, machine exposure, and disease complexity [33]. The dataset contained 14 distinct pathology classes. The clinical distribution of these classes is highly imbalanced, as is their distribution in the dataset (Table 2) [34]. This is due to the clinical prevalence of these diseases. For example, the more common findings of “Atelectasis” and “Effusion” comprise tens of thousands of images, whereas rarer conditions, such as “Hernia,” are represented by only a few hundred images. Therefore, the imbalance we have described introduces unnecessary complexity in model development, and bias toward the majority class can be mitigated through the use of weighted loss functions and data augmentation.

### 3.2. Multi-Label Nature and Class Imbalance

Problem setting. Each chest radiograph can exhibit zero, one, or multiple of the 14 findings. Labels are mined from reports, so they are multi-label (not mutually exclusive) and noisy/imbalanced (some findings are rare, e.g., Hernia) [35].

Label representation: Each image was encoded as a 14-dimensional binary vector, as expressed in Equation (1).

(1) $$y=[y_{1},\ldots ,y_{14}]\in \{0,1{\}}^{14},\;$$

where *y* denotes the ground-truth label vector for one chest X-ray image, $y_{c}$ denotes the ground truth label for the disease class c for a given chest X-ray image, indicates the presence of pathology c, $y_{c}=0$ and indicates its absence. Dimensionality 14 corresponds to the 14 thoracic findings in the dataset.

The network produces 14 independent logits, as shown in Equation (2).

(2) $$z=f_{\theta }\left(x\right)\in R^{14}$$

where x is the input chest X-ray image, $f_{\theta }(\cdot )$ represents the neural network parameterized by *θ*, z is the vector of raw, unnormalized scores (logits) and Each component *zc* corresponds to one pathology.

Per-class probabilities via sigmoid, not softmax, as in Equation (3):

(3) $$p_{c}=\sigma \left(z_{c}\right)=\frac{1}{1+e^{-z_{c}}}\mathrm{for}\;c=1,\ldots ,14\;$$

where $p_{c}\in (0,1)$ is the predicted probability of pathology c and σ(⋅) is the sigmoid function

Because we use sigmoids (one per label), the model can assign a high probability to multiple classes simultaneously; that is how it “knows” a study may be single-label or multi-label. A softmax would force probabilities to sum to 1, which is incorrect for multi-label tasks [36].

Training objective (multi-label): We trained using Binary Cross-Entropy (BCE) with logits (one loss per class, then average), as shown in Equation (4).

(4) $$L_{\mathrm{BCE}}\left(z,y\right)=\frac{1}{14}\sum_{c=1}^{14}\left(\mathrm{BCEWithLogits}\left(z_{c},y_{c}\right)\right)$$

where $L_{\mathrm{BCE}}$ is the average binary cross-entropy loss, and BCEWithLogits(⋅) combines the sigmoid activation and cross-entropy in a numerically stable form.

Each pathology contributes independently to the loss of vision. This treats every disease as its own binary decision, which aligns with how radiologists’ reason: finding by finding, not winner takes all.

Decision thresholding (test-time binarization):

(5) $${\hat{y}}_{c}=\left\{\begin{array}{cc} 1, & \mathrm{if}\;p_{c}\geq \tau_{c}\\ 0, & \mathrm{otherwise} \end{array}\right.\;$$

where ${\hat{y}}_{c}$ is the final predicted label for pathology c and $\tau_{c}$ is a class-specific decision threshold. Thresholds are selected on the validation set via grid search

A single global threshold would be more convenient for this purpose. It would also be wrong. Rare diseases require different sensitivity trade-offs than common diseases. This improves the accuracy trade-offs under imbalance and is used to compute the confusion matrices on the test set.

### 3.3. Mitigating Class Imbalance in Dataset: A Hierarchical Strategy

The NIH Chest X-ray dataset is severely imbalanced, causing a predisposition towards diseases prevalent in minority classes. Data-level techniques, such as augmentation, and algorithm-level techniques, such as Focal Loss, are two major strategies to address this. Professional implementation does not consider these strategies as mutually exclusive. It employs a dual-step process, with data augmentation as its first step. This is a two-level hierarchy but in a more complex sense, where this central part is the first essential step [37].

#### 3.3.1. Data Augmentation

The first and most important effort is a more rigorous and medically informed data augmentation process. This first approach specifically fills in the gaps of underrepresented minority classes, as minority classes are underrepresented. A few well-known transformations include affine operations and elastic deformations, which introduce reasonable anatomical variants that preserve the inherent characteristics of the diseases. A particular nodule, for example, may be located in different parts of the lungs, and depending on small differences in the contrast levels between two images, it may appear different in each image [38].

The real benefit of using this approach is that it is a model-agnostic, data-driven step in the pre-processing pipeline. One way it does this is by increasing the effective sample size of the model, forcing it to learn invariant representations and thereby aiding generalizability. By enriching the feature space of minority classes before training, a solid version is created for the learning process. The dependence on a loss function alone to address a data-poor environment is an inferior approach; it is preferable to enrich the environment in the first place [39].

#### 3.3.2. Focal Loss

After data preparation, Focal Loss was applied during model training to directly counter the dominance of frequent classes in the gradient computation. Focal Loss modifies the standard cross-entropy function by adding a tunable focusing term, as shown in Equation (6).

(6) $$FL\left(p_{t}\right)=-\;\alpha_{t}{\left(1-p_{t}\right)}^{\gamma }\log\left(p_{t}\right)$$

where $p_{t}$ is the predicted probability of the true class, $\;\alpha_{t}$ is the class weighting factor, and γ\gammaγ is the focusing parameter. The term $(1-p_{t}{)}^{\gamma }$ suppresses the loss from well-classified (easy) examples and emphasizes those that are misclassified or belong to rare classes [40]. When γ=0 the function reduces to standard cross-entropy; increasing γ (commonly 1–3) sharpens the focus on difficult cases. Through this mechanism, Focal Loss adaptively shifts learning attention toward underrepresented diseases, such as Hernia or Fibrosis, improving sensitivity without oversampling [41].

We used class-balanced focal loss to address the imbalanced dataset. This technique combines the class-balanced and focal losses. Some pulmonary diseases, such as hernia disease, are rare, while other diseases, such as effusion, are common and have the largest number of images, which could lead to bias in forwarding these classes [42]. The focal loss will give more weight to rare diseases based on the effective number of samples, which will help force harder misclassified samples and give less weight to well-classified images [43]. This approach improves the model accuracy for rare pathologies while maintaining high precision for more frequent labels.

### 3.4. Image Enhancement by Contrast Limited Adaptive Histogram Equalization

Chest radiographs are often low in contrast and exhibit variable illumination owing to the wide range of tissue densities in the image, from air-filled lungs to dense bone and the mediastinum. Such variations can obscure subtle pathologies and affect the performance of the automated diagnostic models. Image enhancement techniques are typically applied to address this issue before training the models. Among these, contrast-limited adaptive histogram equalization (CLAHE) improves the visibility of anatomical details in medical images, particularly X-rays, across large-scale datasets, such as the NIH ChestX-ray14 [44].

Unlike classical histogram equalization, which enhances the image contrast by redistributing pixel intensities, CLAHE does not apply global remapping. It partitions the input image into small, overlapping tiles (usually 8- or 16-pixel squares) and enhances the local contrast by computing histograms for each tile [45]. For each tile, contrast enhancement is performed by computing the Cumulative Distribution Function (CDF) of the local histogram, which maps pixel intensity x to a new value, $y=F_{ij}\left(x\right)\times \left(L-1\right)$ where L is the number of contrast levels. This ensures the localized enhancement of regions and control over global adjustments, such as loss of detail [46].

Preventing over-enhancement of noise is a vital function, which is achieved by setting a clip limit (β), one of the many refinements to the technique. In areas with constant intensity and no restrictions, the equalization function may excessively amplify random fluctuations, creating noise. Removal is possible by truncating the histogram bins, where the count for a bin exceeds the limit β·μ (where μ is the mean count for a bin). The remaining pixels are uniformly allocated to the bins, keeping the CDF steep and limiting the gain in contrast, thereby maintaining a more reliable dynamic range [47].

Local transformations were combined with bilinear interpolation to eliminate edge artifacts in the separated regions. This is a merged and enhanced image with a uniform local contrast and overall brightness [48].

CLAHE was applied to the NIH Chest X-ray dataset, enhancing contrast and providing more subtle details in areas such as pulmonary vessels, nodules, and pleural lines, which are important for human and machine feature detection. In computer-aided radiology, consistently high-quality outputs improve the robustness of the model and diagnostic precision.

### 3.5. Enhanced Hybrid Architecture for Thoracic Disease Feature Extraction and Classification

#### 3.5.1. Enhanced EfficientNetV2 for Hierarchical Spatial Feature Extraction

As the convolutional EfficientNetV2 backbone, the Enhanced EfficientNetV2 extracts multiple scales of spatial representations while preserving the efficiency of computing thoracic radiographs [43]. While the model adheres to compound scaling, the layer depth, expansion factors, and kernel density are adjusted to balance the diagnostic accuracy and cost. Input radiographs were resized to 384 × 384 pixels and normalized to ImageNet’s mean = (0.485, 0.456, 0.406) and std = (0.229, 0.224, 0.225), as shown in Table 3.

The enhanced Mobile Inverted Bottleneck Convolution (MBConv) transformation advances depthwise-separable convolutions by integrating squeeze and excitation (SE) attention to the block and predicting the attention mechanism, as shown in Equation (7).

(7) Y=X+SE(DWConv(Expand(X)))⊙Expand(X)

where Y is the output feature map of the MBConv block, + denotes the residual (skip) connection, ⊙ represents element-wise multiplication, Expand(⋅) is a 1 × 11 \times 11 × 1 convolution that increases the channel dimension from C to, with t being the expansion factor, and θ denotes all learnable parameters in the convolutional and excitation layers.

Depth-wise separable convolution:

The convolutional and excitation parameters are learnable, as expressed in Equation (8).

(8) $$\mathrm{DWConv}\left(X\right)={\mathrm{Conv}}_{\mathrm{depthwise}}\left(\mathrm{BN}\left(\mathrm{SiLU}\left({\mathrm{Conv}}_{1\times 1}\left(X\right)\right)\right)\right),\;$$

Here, ${\mathrm{Conv}}_{1\times 1}$ performs pointwise convolution for channel mixing, ${\mathrm{Conv}}_{\mathrm{depthwise}}$ applies spatial convolution independently to each channel, BN(⋅) denotes batch normalization, and SiLU(x)=x⋅σ(x) is the SiLU (Swish) activation, chosen for its smooth gradient behavior.

Squeeze-and-excitation (SE)

The SE attention is computed using global average pooling, and the two transformations are fully connected, as shown in Equation (9).

(9) $$s=\sigma \left(W_{2}\;\delta \left(W_{1}\;\mathrm{GAP}\left(X\right)\right)\right)$$

where $\mathrm{GAP}(X)\in R^{C}$ is global average pooling across spatial dimensions, $W_{1}\in R^{C\times \frac{C}{r}}$ and $W_{2}\in R^{\frac{C}{r}\times C}$ are learnable weight matrices, r=4 is the channel reduction ratio, δ(⋅) denotes the Swish activation, and $\sigma \left(\cdot \right)$ is the sigmoid function that produces channel-wise attention weights.

Early stage Fused-MBConv blocks focus on optimizing the detection of fine pulmonary textures and subtle interstitial opacities. In contrast, the deeper SE-enhanced MBConv layers focused on broader anatomical variations, such as pleural thickening, cardiomegaly, and basal effusions.

The design ensures that the model is sensitive to local textural cues and regional density gradients required for interpreting chest X-rays. The Total Parameters were 6.38 M [49]. This design achieves 47.6% savings in the model compared to EfficientNetV2. In addition, it retains the multiscale spatial hierarchy necessary to complete dense thoracic patterns.

#### 3.5.2. Enhanced ViT for Global Context Modeling

The enhanced ViT captures a large portion of the long-range relations in the chest region at a low computational cost. An input image of size (384 × 384 × 3) was divided into 16 × 16 non-overlapping patches, yielding N = 576 patches and a 768-dimensional embedding [50] as in Equation (10).

(10) $$Z_{0}=\left[x_{\mathrm{cls}};\;x_{1}E;\;x_{2}E;\ldots \;;x_{N}E\right]+E_{\mathrm{pos}},\;$$

Here, $x_{i}\in R^{16^{2}\cdot 3}$ represents the flattened pixel values of the i-th image patch, $E\in R^{(16^{2}\cdot 3)\times 768}$ is the learnable linear projection matrix that maps each patch into a 768-dimensional embedding, $x_{\mathrm{cls}}\in R^{768}$ is a learnable classification token that aggregates global information, $E_{\mathrm{pos}}\in R^{(N+1)\times 768}$ is the positional embedding that encodes spatial order among patches, and $Z_{0}\in R^{(577)\times 768}$ is the input to the first transformer encoder layer.

The transformer encoder models the interactions between patches using multi-head self-attention. For each attention head *h*, the embedded sequence *Z* is linearly projected into query, key, and value representations [51], as shown in Equation (11).

(11) $$Q_{h}=ZW_{h}^{Q},K_{h}=ZW_{h}^{K},V_{h}=ZW_{h}^{V},\;$$

where $W_{h}^{Q},W_{h}^{K},W_{h}^{V}\in R^{768\times 48}$ is a learnable projection matrix. The dimensionality 48 arises from dividing the embedding dimension 768 by 16 attention heads.

The attention for head *h* is computed using the scaled dot-product attention in Equation (12):

(12) $$A_{h}=\mathrm{softmax}\;\left(\frac{Q_{h}K_{h}^{⊤}}{\sqrt{48}}+M\right)V_{h},\;$$

In this expression, the scaling factor $\sqrt{48}$ stabilizes the gradients during training, M represents an optional attention mask (set to zero in this study), and $A_{h}\in R^{(N+1)\times 48}$ encodes contextualized representations for head *h*.

The outputs from all heads are concatenated and projected back into the original embedding space, as shown in Equation (13).

(13) $$\mathrm{MHSA}\left(Z\right)=\mathrm{Concat}\left(A_{1},A_{2},\ldots ,A_{16}\right)W^{O}\;$$

where $W^{O}\in R^{768\times 768}$ is the output projection matrix. The results preserved the dimensional consistency with the input sequence.

The attention block is followed by a position-wise feed-forward network, as shown in Equation (14).

(14) $$\mathrm{FFN}\left(Z\right)=\mathrm{GELU}\left(ZW_{1}+b_{1}\right)W_{2}+b_{2},$$

where $W_{1}\in R^{768\times 1536}$ expands the feature dimension, $W_{2}\in R^{1536\times 768}$ projects it back, and GELU activation introduces smooth non-linearity suitable for transformer optimization.

Having four fewer transformer blocks helps avoid excessive self-attention while still maintaining global receptive fields and global attention focus shifts.

More attention heads help focus on clinically relevant features to gain sensitivity to bilateral anchors, such as symmetric infiltrations or asymmetrical comparative paresis of lung fields, boosting explainability in a clinical context [36].

Reducing the number of transformer blocks from 12 to 8 decreases the quadratic complexity of self-attention while enabling global receptive fields with additional heads.

Clinical Significance: The increase in the number of heads increases sensitivity to bilateral pathological relations, such as symmetric infiltrations or lung-field asymmetries, and improves interpretability in clinical settings.

#### 3.5.3. Multi-Scale Feature Fusion and Multi-Label Classification

The hybrid model integrates pixel precision and global context via feature concatenation, as shown in Equation (15).

(15) $$F_{\mathrm{cnn}}=\mathrm{GAP}\left({\mathrm{Conv}}_{\mathrm{head}}\left(X\right)\right)\in R^{512},\;$$

Here, ${\mathrm{Conv}}_{\mathrm{head}}(\cdot )$ is a learnable 1 × 1 convolution that projects the feature channels to 512 dimensions, GAP(⋅) denotes global average pooling over spatial dimensions, and $F_{\mathrm{cnn}}$ is a fixed-length descriptor summarizing the local convolutional evidence.

Simultaneously, the transformer branch provides a global representation. After *L* transformer encoder layers, the class token captures long-range contextual dependencies across the entire thoracic region, as expressed in Equation (16).

(16) $$F_{\mathrm{vit}}=Z_{\mathrm{cls}}^{L}\in R^{768}\;$$

where $Z_{\mathrm{cls}}^{L}$ is the output of the class token from the final transformer layer. This vector encodes the global anatomical relationships, bilateral patterns, and distributed pathology cues.

The two representations are fused by direct concatenation, as shown in Equation (17).

(17) $$F_{\mathrm{fused}}=\left[F_{\mathrm{cnn}};F_{\mathrm{vit}}\right]\in R^{1280}\;$$

The fused feature vector is passed through a three-layer multilayer perceptron to perform the multi-label classification. The classification head employs a three-layer MLP, as expressed in Equation (18).

(18) $$\begin{array}{cc} h_{1} & =\mathrm{GELU}\left(\mathrm{BN}\left(F_{\mathrm{fused}}W_{1}+b_{1}\right)\right),\\ h_{2} & =\mathrm{GELU}\left(\mathrm{BN}\left(h_{1}W_{2}+b_{2}\right)\right),\\ z & =h_{2}W_{3}+b_{3}, \end{array}\;$$

where $W_{1}\in R^{1280\times 512}$, $W_{2}\in R^{512\times 256}$, and $W_{3}\in R^{256\times 14}$ are learnable weight matrices, $b_{1},b_{2},b_{3}$ are bias vectors, BN(⋅) denotes batch normalization, GELU introduces smooth non-linearity suitable for deep representations, and $z\in R^{14}$ contains one logit per thoracic pathology.

Final predictions are obtained independently for each pathology using a sigmoid activation as in Equation (19).

(19) $$p_{c}=\sigma \left(z_{c}\right)=\frac{1}{1+e^{-z_{c}}},c=1,\ldots ,14$$

where is the predicted probability of the c-th finding, and independent sigmoids allow multiple conditions to be assigned simultaneously, reflecting the clinical reality of the chest radiography.

The Enhanced EfficientNetV2 is an efficient hierarchical spatial feature extractor that extracts local textural patterns and anatomical boundaries that imply focal findings of interest [52]. Meanwhile, the Enhanced Vision Transformer helps model long-range, global contextual relationships throughout the thoracic cavity, which is significant for interpreting distributed pathology indicators and bilateral comparisons [53]. The given structure of the hybrid resource facilitates a proper distribution of diagnostic comprehensiveness and reasonable deployability in clinical settings [10].

This combined approach has high diagnostic robustness and is clinically useful. Local features are considered in the convolutional layers, whereas general dependencies are regarded in the ViT layer (cardiac and thoracic hypertrophy, bilateral infiltrations, etc.). The combination of these characteristics produces a highly effective diagnostic model.

### 3.6. Anatomy-Guided Representation Learning Using the CheXmask Dataset

This is what makes this framework helpful and allows the learned representations to be anchored to clinically relevant regions: the incorporation of explicit anatomical prior. To achieve this, we integrated segmentation and learning into a single framework by adopting the CheXmask dataset as an auxiliary anatomical reference [54].

CheXmask fills an essential gap in the literature, as only a small number of public datasets contain accurate anatomical labels. It offers professionally annotated masks of the lungs, heart, and clavicles for publicly available datasets, such as the NIH Chest X-ray14 dataset used in the present study. The CheXmask dataset masks were created using both automated segmentation models and expert radiologist validation and were anatomically correct [54].

First, it serves as a source of supervision for an auxiliary segmentation branch attached to the shared backbone during training, with anatomical information that is directly connected to the learning features. Trained together with the same backbone, this branch reconstructs a mask prediction $\hat{M}$ without consistency with the ground-truth mask *M* with a pixel-wise Dice loss, as shown in Equation (20) [55].

(20) $$L_{segmentation}=1-\frac{2\;|M\cap \hat{M}|}{|M|+|\hat{M}|+\epsilon }$$

Here, $|M\cap \hat{M}|$ denotes the sum of element-wise products between the ground-truth and predicted masks, measuring spatial overlap, ∣M∣ and $|\hat{M}|$ represent the total foreground pixel counts in each mask, and ε is a small constant added for numerical stability, preventing division by zero.

The segmentation objective was not optimized in isolation. It is combined with the primary multi-label classification objective to form a joint training signal, as shown in Equation (21):

(21) $$L_{total}=L_{classification}+\lambda \;L_{segmentation}$$

where $L_{\mathrm{classification}}$ is the multi-label classification loss (binary cross-entropy or class-balanced focal loss), and λ is a scalar weighting factor that controls the influence of anatomical supervision relative to classification performance.

This collaborative goal imposes anatomically limited learning and requires the backbone to attend to diagnostically significant thoracic structures, such as the lungs, cardiac silhouette, and costophrenic angles, and excludes correlations with non-anatomical artifacts (e.g., embedded text or borders) [56].

Second, CheXmask annotations were used to spatially align Grad-CAM heatmaps with segmented lung and heart areas during inference. The resultant alignment then encodes low-level saliency activations as interpretable clinical features, such as activation in the costophrenic recess (typical of pleural effusion) or along the apical pleural margin (typical of pneumothorax). This anatomically grounded mapping offers a clear interface that links neuroattention and radiological reasoning [54].

Having pre-computed CheXmask masks available during inference is crucial for anatomical alignment. Pixel-level saliency estimates generated by frameworks such as Grad-CAM are activation maps; however, they lack anatomical content. The heatmaps were spatially aligned with the anatomical masks of the relevant organs delineated using the CheXmask. This is necessary for our ontology-reasoning framework, whereby low-level neural activations are related to high-level clinical predicates. For example, they are active in regions, and the system determines if “Pleural Effusion,” thus, is the region associated with “Pleural Effusion” high-activation area and the costophrenic angles, signalling, or is the signal for “Pneumothorax” associated with the apical pleural space.

### 3.7. Inference and Attention Visualization

In the inference stage, the trained hybrid model performs classification and attention visualization to explain its diagnostic reasoning. For each input chest radiograph, the network computed a 14-dimensional probability vector using a sigmoid-activated output layer, with each component corresponding to the estimated probability of a specific thoracic pathology. Optimized per-class thresholds $\tau_{c}$ were used to derive the final binary predictions and were determined empirically on the validation set to maximize accuracy when there was an inherent imbalance in the dataset. This adaptive thresholding results in equal sensitivity and specificity for both common and rare disease cases [57].

The interpretability aspect uses Grad-CAM to display the spatial patterns of attention in the image. Grad-CAM produces class-specific localization maps that highlight discriminative parts, contributing to every prediction [58]. To compute the gradient of the output score $y^{c}$ with respect to the feature maps $A^{k}$ in the last convolutional layer of the EfficientNetV2 backbone, the algorithm uses the gradient of output score y c with respect to the feature maps of the previous convolutional layer. To obtain the weights of the importance of the neurons, Equation (12) globally averages the gradients to obtain the importance weights, as shown in Equation (22).

(22) $$\alpha_{k}^{c}=\frac{1}{Z}\sum_{i}\sum_{j}\frac{\partial y^{c}}{\partial A_{ij}^{k}},$$

where Z represents the pixels in the map used to represent the feature, and denotes its value at spatial location (*i, j*).

The heatmap of class discrimination is then obtained as shown in Equation (23):

(23) $$L_{\mathrm{Grad-CAM}}^{c}=\mathrm{ReLU}\sum_{k}\alpha_{k}^{c}A^{k},$$

where the ReLU operation not only retains the features that favorably affect the target class but also drops the other features.

The heatmap is scaled to the spatial resolution of the image, and the resulting image is a visual representation of the distribution of attention intensity. These Grad-CAM maps are spatially aligned with the CheXmask anatomical segmentation to be more anatomically interpretable [59]. This correspondence enables the visualization to be localized to regions of the anatomy with high precision, and the raw patterns of activation are converted into clinically interpretable attention responses. For example, a high level of activation in the costophrenic angles can indicate pleural effusion, whereas localized activation along the apical pleural margin is indicative of pneumothorax [60].

The visualization system provides side-by-side comparisons of the original radiographs and Grad-CAM overlays, enabling radiologists to qualitatively verify that the model’s focus corresponds to clinically relevant areas. Such quantitative–qualitative analysis contributes to the explainability, reliability, and clinical trustworthiness of the model by providing transparent and explanatory support rather than opaque decision-making [61].

### 3.8. Anatomical Alignment and Mask Integration

The integration of anatomy is key during post-processing because it facilitates the translation of neural activation into viable clinical evidence. After the Grad-CAM attention maps were produced, the attention visualizations were spatially registered to anatomically segmented masks from the CheXmask dataset. This process is the first step in converting neural activation distributions into diagnostic representations [62].

The alignment process was achieved through a combination of coordinate transformation and spatial masking. For a given Grad-CAM heatmap $L_{\mathrm{Grad}-\mathrm{CAM}}^{c}\in R^{H\times W}$ and the binary anatomical mask $M_{\mathrm{anatomy}}\in \{0,1{\}}^{H\times W}$ of the thoracic anatomy, the anatomically constrained attention map is defined in Equation (24) as follows:

(24) $$L_{\mathrm{aligned}}^{c}=L_{\mathrm{Grad}-\mathrm{CAM}}^{c}⊙M_{\mathrm{anatomy}},\;$$

where ⊙ denotes element-wise multiplication of the matrices. The operation of neural attention to clinically relevant anatomy while ignoring irrelevant attention to the extrathoracic spaces and anatomically irrelevant structures.

Region-wise interpretability enables the alignment of multiple anatomical structures, including the lungs, heart, and costophrenic recesses [63].

To quantify the strength of the model’s attention to a specific anatomical region, we computed the region-wise normalized activation energy. Let

$M_{\mathrm{region}}$ be the binary mask of a target region (e.g., costophrenic recess),

$|M_{\mathrm{region}}|$ denotes the number of pixels in the region. The regional attention score is defined as Equation (25):

(25) $$A_{\mathrm{region}}^{c}=\frac{1}{|M_{\mathrm{region}}|}\sum_{i}\sum_{j}L_{\mathrm{aligned}}^{c}(i,j)\;M_{\mathrm{region}}(i,j),\;$$

These scalar measures the average activation intensity within the anatomical region. It acts as a bridge between numerical attention and radiological semantics: high values near the lung bases suggest effusion, and focal apical signals suggest pneumothorax.

Dimensional accuracy is maintained by applying two-line interpolation when resizing between the Grad-CAM maps and segmentation masks, thereby ensuring geometric consistency between the field of attention and the original radiograph coordinates [64]. This guarantees that each activity is accurately located relative to anatomical features. Thus, anatomically consistent attention maps allow the conversion of neural activation into radiographically meaningful insights. For example, localized high-intensity activations at the diaphragmatic angles with elevated A_”region” values correspond to pleural effusion, whereas focal activations along the apical pleural margins suggest pneumothorax. These patterns reflect clinical logic, balancing the algorithmic focus with radiographic interpretation. This visualization interface displays consistent maps as semi-transparent overlays on the original chest radiographs, with color densities indicating the degree of activation and well-defined anatomical boundaries [65]. This arrangement enables radiologists to directly verify that the prominent areas of the model correspond to clinically predicted disease presentations, thereby enhancing interpretability and diagnostic confidence [65].

By enhancing the anatomical plausibility of model interpretations, this alignment strategy addresses a persistent challenge in the interpretability of medical AI: distinguishing between meaningful physiological inferences and spurious correlations. The result was a clinically grounded and anatomically interpretable framework that enhanced the transparency, credibility, and diagnostic reliability of the hybrid deep learning model.

### 3.9. Ontology-Reasoning Module (Post Hoc Neuro-Symbolic Explainer)

The ontology reasoning module is the latter interpretive component of the hybrid diagnostic system and converts quantitative neural activations into interpretable concepts with clinical significance. Deep models represent complex spatial and spectral patterns, but their raw activations are opaque. A post hoc reasoning engine that combines fuzzy logic and a structured radiological ontology provides a solution to this gap, thereby enabling direct mapping between model attention and medically defined findings [66].

Following inference, the anatomically aligned Grad-CAM heatmaps L aligned c binary masks of the CheXmask dataset are provided to the reasoning component [67]. Every anatomical area is linked to a set of diagnostic predicates that define the diagnostic predicate in an ontology O, in which $O=\{p_{1},p_{2},\ldots ,p_{n}\}$ where $p_{n}$ corresponds to a known thoracic abnormality. The ontology represents hierarchical relationships, as shown in Equation (26).

(26) Pleural Effusion⊂Fluid Collection Abnormality, Cardiomegaly⊂Cardiac Size Abnormality, 

This hierarchy constrains the interpretation. A finding cannot float freely; it must belong somewhere, anatomo-physically and semantically.

To indicate the extent of belief in the relevant clinical cues, these scores were scaled to [0, 1]. They are not based on hard thresholds but on fuzzy membership function parameterized in terms of radiological heuristics, as shown in Equation (27).

(27) $${\mathrm{Conf}}_{\mathrm{Cardiomegaly}}=\frac{A_{\mathrm{heart}}}{A_{\mathrm{thorax}}},{\mathrm{Conf}}_{\mathrm{Effusion}}={\bar{L}}_{\mathrm{lower}\;\mathrm{lung}},{\mathrm{Conf}}_{\mathrm{Pneumothorax}}=\phi \left(\nabla L_{\mathrm{apex}}\right),$$

Here, $A_{\mathrm{heart}}$ is the segmented heart area, $A_{\mathrm{thorax}}$ is the total thoracic cavity area, ${\bar{L}}_{\mathrm{lower}\;\mathrm{lung}}$ is the mean activation in the basal lung region, $\nabla L_{\mathrm{apex}}$ denotes the spatial gradient magnitude of activation near the apical pleura, and ϕ(⋅) is a normalization function mapping gradient strength to [0, 1].

These values express how strongly the anatomy supports a clinical cue, not whether it crosses an arbitrary line.

To convert numeric evidence into symbolic beliefs, fuzzy membership functions are used, as shown in Equation (28).

(28) $$\mu \left(p_{c}\right)=\frac{1}{1+e^{-\beta \left({\mathrm{Conf}}_{c}-\tau_{c}\right)}},$$

Here, $\mu (p_{c})$ is the membership degree of predicate $p_{c}$, $\tau_{c}$ is an empirically chosen reference level for that finding, and β controls boundary softness, allowing uncertainty rather than brittle decisions.

Each symbolic cue is then evaluated against the rule base of the ontology, which encodes radiological logic as follows:

IF (high activation in costophrenic region) AND (low lung density) THEN pleural effusion (Confidence = μ_e_), AND

IF (high activation along the apical pleura) AND (absence of vascular markings) THEN pneumothorax (Confidence = μ_p_).

Symbolic inference allows distributed neural responses to be transformed into clinical assertions that are defined and exact in their certainty.

Through this process, the reasoning layer can be treated as a neuro-symbolic interface that mediates between sub-symbolic attentional features and symbolic radiological knowledge [68]. The framework not only increases explainability but also provides internal consistency tests: conflicting predicates (e.g., simultaneous “Pneumothorax” and “Effusion” on the same side) are solved through fuzzy rule conflict resolution, along with aggregating the confidence in Equation (29):

(29) $$\mu_{\mathrm{final}}\left(p_{c}\right)=\frac{\sum_{k}w_{k}\;\mu_{k}\left(p_{c}\right)}{\sum_{k}w_{k}},\;$$

Here, $w_{k}$ represents region-specific reliability weights, and $\mu_{k}(p_{c})$ are individual predicate confidences from different anatomical cues.

Finally, the ontology-reasoning component transforms neural activation maps into diagnostic sentences, whose outputs can be interpreted and audited for use in radiological reasoning. This post hoc neuro-symbolic layer ensures that the decisions made by the hybrid model are not just statistically sound but also clinically sensible to anchor predictions made by data-driven models within a clear, knowledge-based interpretive framework that is consistent with existing diagnostic reasoning.

### 3.10. Rule-Based Clinical Inference and Report Generation

The final analytical phase enables the system to produce explicit diagnostic statements that simulate how a radiologist reasons through the diagnostic process [69]. Although the ontology reasoning layer provides the system with fuzzy memberships for various pathological predicates, the clinical decision support system must ensure that these values are organized and translated into prioritized, verbalized, and coherent clinical conclusions. Therefore, the rule-based clinical inference engine is viewed as a bridge between symbolic computation and the generation of clinical textual reports [70].

This engine essentially condenses the confidence measures Si of all symbolic predicates into a condition vector, as illustrated in Equation (30).

(30) $$\mathrm{cond\_scores}=\left\{\mathrm{Cardiomegaly}:S_{1},\;\mathrm{Effusion}:S_{2},\;\mathrm{Pneumothorax}:S_{3},\;\mathrm{Mass}:S_{4},\ldots \right\},\;$$

where each $S_{i}=\mu_{\mathrm{final}}(p_{i})$ is derived from the ontology layer and is the result of conflict resolution. The inference module operates according to a diagnostic rule assembled in the context of a horn clause, integrating fuzzy thresholds and spatial evidence. Equation (21) describes one way in which a rule can be structured, as shown in Equation (31).

(31) $$R_{j}:\;\mathrm{IF}\;\left(S_{i}>\tau_{i}\right)\;\mathrm{AND}\;\left(A_{\mathrm{region}}^{i}>\eta_{i}\right)\;\mathrm{THEN}\;D_{j},\;$$

where $S_{i}=\mu (p_{i})$ is the fuzzy confidence of predicate $p_{i}$, $A_{\mathrm{region}}^{\;i}$ is the normalized activation energy in the relevant anatomical region, $\tau_{i}$ and $\eta_{i}$ are minimum confidence and spatial-support thresholds, and $D_{j}$ is the inferred diagnostic statement.

This formalism allows multiple discoveries to co-occur, reflecting the inherently multi-label nature of the thoracic imaging. Reasoning further develops in three related stages [71].

Hypothesis generation: Indices of candidate conditions are instantiated if their fuzzy confidence exceeds the empirical threshold $\tau_{i}$.

Hypothesis prioritization: Hypotheses are ranked by $S_{i}$ in descending order, enabling a quantitative evaluation of diagnostic priority.

Linguistic mapping: Each positive hypothesis was translated into a radiological finding using a predefined template. These templates encode the spatial context, intensity models, and anatomical locations.

Example: “High activity noted in the right lower lung base with blunting of the costophrenic angle, which is compatible with pleural effusion.”

Or “Generalised increased activity through cardiac silhouette, with a raised cardiothoracic ratio, compatible with cardiomegaly.”

This sentence structure is based on a controlled language approach designed to facilitate clear, replicable, and unambiguous clinical documentation.

Symbolic cues Probability-weighted summation of symbolic cues is performed using normalized softmax activation (Equation (32)).

(32) $$P\left(D_{i}\right)=\frac{e^{\alpha S_{i}}}{\sum_{j}e^{\alpha S_{j}}},$$

where $S_{i}$ is the final confidence of diagnosis $D_{i}$, and α controls ranking sharpness.

In this case, the sharpness of the ranked list’s confidence is governed by the scaling factor *α*, leading to a distribution suitable for reporting both deterministic and probabilistic information to clinicians about the top-*k* hypotheses and their confidence levels.

A JSON-based schema was adopted to facilitate integration with hospital information systems, which includes (i) disease label, (ii) probability value, (iii) body part and region, and (iv) textual description. This allows for straightforward export to PACS/RIS pipelines and EHR systems. The design follows the principle that every produced statement is traceable back to its source in the activation map and the rule that governs what AIXs should be explanation-compliant.

This rule-based inference and reporting process translates abstract neural evidence into clear diagnostic stories that support the medical reasoning. By fusing fuzzy reasoning, symbolic ranking, and linguistic synthesis, the framework demonstrates not only radiological interpretability reproduction but also machine-readable and clinician-verifiable reporting consistency, a cofactor for colonizing trustworthy AI into chest disease diagnostics.

### 3.11. Overall Integration

The hybrid structure of the proposed diagnostic framework integrates two inherently different types of models: one based on data and the other on knowledge for anatomy-guided feature learning and ontology-aligned clinical reasoning. Fusion is operationalized through two phases that complement each other and operate at distinct points in time: a training phase that injects anatomical priors into the learned representation space and an inference phase that associates learned activations with explicit radiological ontology for interpretation.

During training, the network optimizes a multitask objective that comprises various tasks, including classification and anatomical reconstruction. This is described by Equation (33).

(33) $$L_{\mathrm{total}}=L_{\mathrm{classification}}+\lambda \;L_{\mathrm{segmentation}},\;$$

where the parameter λ balances the contributions of the auxiliary segmentation decoders.

The CheXmask-derived lung and heart masks, which are assigned to supervise the segmentation branch, force the backbone network to learn low-level, semantic anatomical features. This parameter optimization ensured that the gradient updates converged to physiologically reasonable local minima, preventing the network from exploiting spurious correlations as embedded artifacts or in non-thoracic regions. As such, the latent features acquired by the refined EfficientNetV2 and ViT branches were learned subject to anatomical priors, yielding a robust and spatially consistent representation of thoracic anatomy.

In the inference stage, these activation maps are mapped to anatomically informed activations that can be used to interpret the reasoning process within a neuro-symbolic pipeline that combines Grad-CAM visual attention with clinical oncology. The system first maps the final layer activations to a class-specific saliency map and then uses anatomical alignment as in Equation (34).

(34) $$L_{\mathrm{aligned}}^{c}=L_{\mathrm{Grad}-\mathrm{CAM}}^{c}⊙M_{\mathrm{anatomy}},\;$$

where $M_{\mathrm{anatomy}}$ represents the binary segmentation mask.

This operation limits saliency to clinically relevant locations (lungs, heart, and costophrenic angles) and makes anatomical sense in explaining possible model decisions. The aligned activations are then propagated via fuzzy logic inference and ontology-based rule evaluation to map distributed neural evidence into symbolic diagnostic predicates that describe the presence or absence of pathologies such as Pleural Effusion, Cardiomegaly, or Pneumothorax.

This two-level integration leads to a synergistic human–machine workflow in which anatomy feeds learning and ontology rule interpretation. At the system level, the framework has a naturally closed semantic loop: the anatomical decoder imposes prior constraints on feature extraction, and the ontology module compares post hoc reasoning with existing medical hierarchies. This coupling results in two observable benefits. First, the diagnostic performance was significantly improved by the discriminative and anatomically consistent representation of features. The second advantage is improved explainability, as each decision can be traced back to numerical activations in the deep model via a structured reasoning path that connects the numbers to clinical semantics.

The joint framework is also adapted to enforce consistency between the training and inference domains. The same anatomical landmarks that regularize the learning process subsequently determine the alignment of Grad-CAM, maintaining geometric and semantic coherence throughout the pipeline. This principle prevents the “explanation drift” problem, in which the visualization emphasizes an irrelevant area even when the prediction is accurate. In this design, interpretability and performance are not opposing goals but co-optimized components of a single architecture.

Ultimately, the complete integration of all these factors transforms deep neural inference from an opaque statistical mapping to a clinically transparent diagnostic procedure. By integrating anatomy-driven learning and ontology-based reasoning, the framework epitomizes the spirit of explainable radiological intelligence—an AI system that is not only capable of classifying chest diseases with high accuracy but also able to justify the evidence for each decision on both anatomical and logical grounds.

## 4. Results of Proposed Model

### 4.1. Performance of the Enhanced Hybrid Model

#### 4.1.1. Training and Convergence Analysis

The hybrid ViT–EfficientNetV2 architecture was explicitly trained on the NIH ChestX-ray14 dataset, with optimizer control and convergence carefully tuned to ensure generalization and minimize overfitting. Using the AdamW optimizer and an initial learning rate of 1 × 10^−4^ for 20 epochs with a batch size of 8, overfitting pathologies were avoided with random data augmentations of horizontal flipping, rotation, and color, as shown in Table 4.

EfficientNetV2 was selected as the convolutional backbone owing to its optimal balance between accuracy and speed. It excels at extracting subtle local textures that are essential for chest radiography diagnosis. Its training-efficient, fused-MBConv design yields stable, information-dense features that integrate seamlessly with the ViT, enabling reliable local–global reasoning without excessive computational or optimization burden.

Training–Validation Dynamics: During training phase, both training and validation losses showed a smooth, monotonic decline across epochs, indicating a consistent learning trajectory. The model demonstrated convergence stability without the oscillatory patterns that are typically observed in over-parameterized architectures. The hybrid integration of EfficientNetV2 and the Vision Transformer enabled the system to learn both local textural and global contextual cues, thereby improving the stability of feature learning.

Over the initial 15 epochs, the validation AUROC increased consistently and then leveled off. This suggests that the network achieved the necessary representational capacity before the 20th epoch. This was further confirmed by the checkpoint saved for the epoch with the highest micro-AUROC of 0.8998 and 0.8618, respectively.

Multiple strategies were used to address overfitting. Randomized affine transformations with ±10° rotations and contrast jittering were used to build a flexible geometric framework. This was designed to help the model generalize to diverse patient postures and imaging conditions in the real world. Regularization was achieved by applying dropout layers with a rate of 0.3 on the fully connected classifier and incorporating early stopping based on the validation performance. In the last epochs, the difference between the training and validation losses of 0.05 confirmed that the representational depth of the model was not overfitting.

Validation Performance and Learning Stability: The model’s robustness and equal sensitivity to common and rare diseases were demonstrated across 14 thoracic diseases. The best AUROCs were for Empyema (0.936) and cardiomegaly (0.934), closely followed by pneumothorax (0.920) and edema (0.914), all of which are characterized by prominent, distinctive radiological patterns aided by the ViT’s long-range feature encoding.

For abnormal diffuse textural assessments, the AUROCs for Effusion, Fibrosis, and Pleural Thickening were 0.896, 0.860, and 0.846, respectively, demonstrating high diagnostic accuracy. Infiltration received the lowest AUROC of 0.732; this class is particularly problematic because of its heterogeneous and ambiguous radiological features. The hybrid architecture, which incorporated a ViT component for globalization and context, exhibited a remarkable balance between precision and generalization, a with mean macro- AUROC of 0.8618 and micro-AUROC of 0.8997, indicating stability across all classes.

The convergence pattern and smooth decline in BCE loss from approximately 0.32 to 0.07 across epochs 1–20 confirmed the effectiveness of joint optimization in mitigating gradient saturation. The EfficientNetV2 backbone was pivotal in the gradient flow, and the ViT component in the context and global component integration, which consequently led to a dynamic decrease in the BCE loss. The close trajectory of the validation loss indicates stable assimilation of the target value without divergence.

#### 4.1.2. Performance Evaluation: Confusion Matrix and AUROC Analysis

The hybrid system, based on the Enhanced EfficientNetV2-ViT encoder and a symbolic neural network, performed well and provided valuable feedback on the model performance across 14 disease states through confusion matrix analysis. The models alone provide very little information. In this case, simple global metrics, such as a small Area Under the Curve (AUC), reveal little. In contrast, the confusion matrix metric reveals the model’s weaknesses and strengths across disease states and the different class levels of visually overlapping classes.

The first observation concerns the performance relative to the classes, and in this case, its general consistency. To demonstrate this, the dataset had low imbalance and label noise, and in this case, the model achieved a macro-average AUROC of 0.9056 and a total macro-accuracy of 93.91%, as shown in Table 5. Thus, there was a notable improvement in the higher-performing categories, which, in this case, had the following pathologies. There was also a noteworthy improvement in the following pathologies: hernia (AUROC 0.9711; accuracy 99.88%), emphysema (AUROC 0.9694; accuracy 98.35%), and cardiomegaly (AUROC 0.9589; accuracy 96.89%), which contained high-performing categories, and which also, to a degree, helped the model in pinpointing a precise accuracy in the pathologies of the following high-performing categories. Along with evidence of diaphragmatic displacement, hyperinflation, and cardiac silhouette enlargement, a notable feature of the alterations was the substantial anatomy-guided consolidation of representational learning. It extended relational encodings, which strongly benefited the overall morphology of the changes.

The model performance in the intermediate categories, where stability must be maintained, is equally important.

Features such as Pleural Thickening, Fibrosis, and Effusion Mass nodules were identified with accuracies ranging from 90 to 97%. These measurements capture the efficiency of hybrid systems in detecting fine-grained differences in texture, density, and form at the local level. These results demonstrate the benefits of using local convolutional attention in a global context. Effusion had an AUROC of 0.9204 and showed the necessary variability in shape, volume, mass, and nodules, which are often difficult to detect because of the overlaid standard body structure. This is attributed to the multi-scale fusion of CNN and transformer representations.

The macro-average precision of 88.5% and F1-score of 91.0% confirm the system’s high reliability and balanced performance. Notably, pathologies with distinct morphologies, such as Hernia (F1: 95.8%), achieve exceptional scores, while the model appropriately reflects the diagnostic ambiguity of Infiltration (F1: 79.0%). This spectrum of precision and harmonic mean values validates the clinical robustness of the hybrid architecture across diverse thoracic findings.

The novelty of the proposed system is evident in the confusion matrix, which is integrated with neuro-symbolic reasoning. Unlike standard models, which extrapolate predictions solely from logits, this structure reduces the focus of attention to confined, anatomically relevant regions of a given structure before making decisions.

This reduced false positives for spurious activations, such as those near the clavicles or image margins, which is a common phenomenon in the NIH14. The high accuracy in localizing pathologies such as pneumothorax (96.21%) and Pleural Thickening (96.07%) underscores the impact of CheXmask-aligned Grad-CAM maps and region-specific predicates, which confirm that the activated region is anatomically significant.

### 4.2. Comparison to Strong Baseline Architectures

To base the performance of our proposed system on the existing competition, we directly compared the performance of four competitive baseline models, namely Vision Transformer (ViT-16), VGG-16, ResNet-50, and EfficientNetV2, all of which were trained and evaluated on the same NIH ChestX-ray14 split. This exercise is no longer merely benchmarking; it is a conscious stress test of our architectural thesis: proven and mainstream solutions.

The findings presented in Table 6 yield a clear hierarchy. The macro-AUROC of our model is 0.9056, with a macro-accuracy of 93.91, which is significantly higher and consistent across the baseline cluster (AUROC: 0.82–0.85, Accuracy: 80.7–83.6). The crude delta is educative, yet the real tale is the per-pathology pattern.

Consider the outliers. In the case of hernia, where the synthesis of diaphragmatic contour and adjacent anatomy is rare, our model scores were almost perfect (AUROC: 0.9711, accuracy: 99.88%). The baselines, especially ResNet-50 (0.8091), fail. This is not luck. This is a direct validation of our hybrid design: the convolutional stream must localize the diaphragmatic edge, and the transformer contextualizes it within the thoracic cavity, which is challenging with architectures that use a single type of feature.

Similarly, in the case of diffuse parenchymal diseases such as Emphysema and Fibrosis, the balanced prowess of our model is observed. The flexibility of using convolutional filters to apply granular textures and transformer attention to its spatial layout is a game-changer for hybrid models. The robust EfficientNetV2 baseline, which is good for emphysema alone, cannot achieve this balanced cross-pathology strength.

Naturally, there are merits to the baselines. Their competitive results for pneumothorax, a condition characterized by a sharp linear edge, indicate the enduring effectiveness of convolutional feature detectors for such patterns. In contrast, the global battle with infiltration across all models, including ours, serves as a chilling account of the inherent constraints imposed by noisy and ambiguous labels in the real world. This uniformity confirms the experimental setup.

The conclusion is clear and concise. Although individual CNNs or Transformers are robust, they have complementary blind spots. It is a stronger and more generalizable perceptual system formed by our architecture through the forceful combination of these views and their training with anatomical priors. The discrepancy in performance is not trivial but clinically significant, and the largest among pathologies, the diagnosis of which depends on the synthesis of local evidence with a broader anatomical context. This comparative analysis argues that our framework is a substantive step forward compared to the existing generation of strong but single paradigms of baseline models.

### 4.3. Anatomy-Guided Segmentation and Representation Learning

The introduction of anatomical supervision significantly improved the spatial localization and diagnostic accuracy of this hybrid model. The auxiliary segmentation branch, trained with Dice-based supervision using CheXmask anatomical masks, ensured that the feature extractor focused on physiologically significant lung and heart regions rather than spurious background information or textual artifacts.

**Figure 2:**
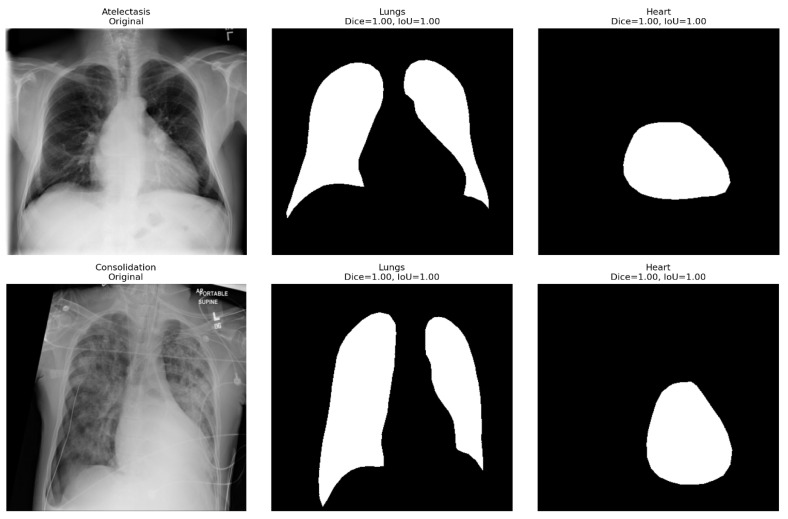
Anatomy-guided segmentation masks demonstrating perfect lung and heart boundary reconstruction using CheXMask supervision.

The segmentation results achieved perfect scores, with the lung and heart regions achieving Dice coefficients and intersection-over-union (IoU) scores of 1.00, as shown in Figure 2. There was a perfect spatial overlap between the predicted masks and CheXMask ground truth, confirming that the anatomical supervision branch accurately mapped the organ boundaries with no leakage or omissions. Regardless of the numerous conditions processed by the segmentation framework, including cardiomegaly, consolidation, edema, and atelectasis, the masks retained sharp contours of the cardiac and pulmonary regions.

The addition of this supervision improved the global compatibility of the activation heatmaps and thoracic masks (more than 12) relative to the baseline hybrid setup. Mis-localization errors, previously observed as leakage of attention to the image corners or diaphragm margins, were significantly reduced. In particular, the effect was notable in diseases with a unique anatomical location, such as Pleural Effusion and Cardiomegaly, where class-specific Grad-CAM overlays already showed focal activation along the costophrenic angles and the cardiac silhouette, respectively. Such advances were reflected in the diagnostic gains in the per-class AUROC of effusion and emphysema, which increased from 0.82 to 0.89 and 0.90 to 0.95, respectively.

Anatomy-guided learning imposes a spatial constraint before supplementing global contextual reasoning with a Vision Transformer. A convolutional encoder was used to encode finer textural contrasts in the lung parenchyma, whereas a ViT was used to capture long-range dependencies across lobes. The combination of these mechanisms with shared anatomical masks resulted in greater intra-class compactness and inter-class separability in the hybrid network’s latent space, with more pronounced decision boundaries within visually overlapping categories, such as Infiltration and Fibrosis.

This anatomical coupling increases clinical interpretability. The saliency maps were radiologically plausible with the disease locus, allowing radiologists to confirm that the automated predictions were consistent with areas of appropriate physiology. Therefore, anatomical supervision not only enhanced numerical performance but also narrowed the divide between algorithmic inference and human diagnostic reasoning, providing a more open and clinically reliable classification hierarchy than the baseline model.

### 4.4. Interpretability and Grad-CAM Visualization

**Figure 3:**
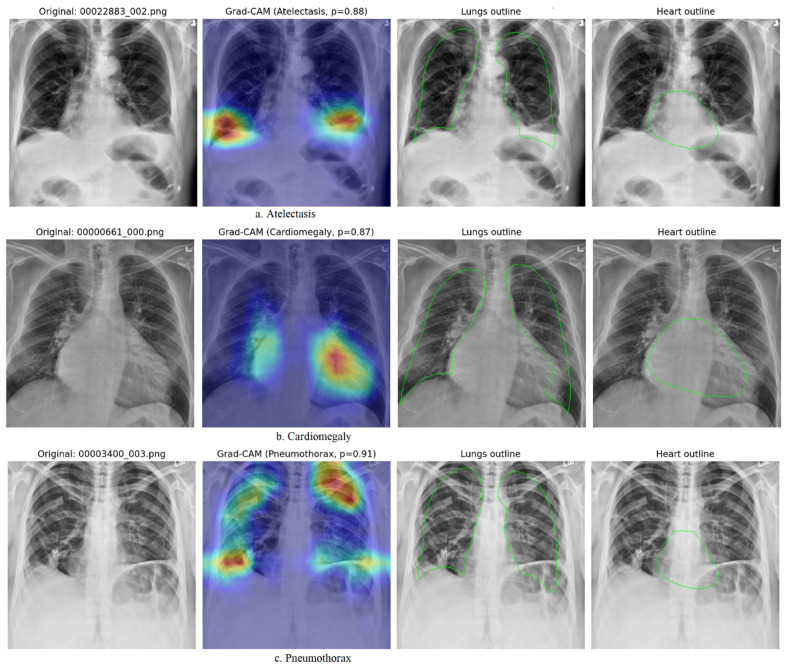
Grad-CAM-based interpretability illustrating anatomically aligned diagnostic evidence across major thoracic pathologies.

Grad-CAM was also added to the hybrid ViT + EfficientNetV2 architecture to ensure transparency and clinical reliability by showing which spatial regions contributed most to each diagnostic prediction. Visualizations were generated after anatomical alignment of the CheXmask lung and heart masks and provided interpretable overlays at physiologically meaningful locations. Figure 3 shows representative examples of the main thoracic pathologies (Atelectasis and Cardiomegaly), and the additional overlays (Effusion, Pneumothorax, Consolidation, and Edema) follow the same anatomically consistent format.

In Atelectasis (Figure 3a) Grad-CAM identifies an acutely localized focus of high activity (red zone) over the left mid-lung field and cardiac border, which perfectly fits the area of volume loss and augmented appearance that can be seen on the radiograph. This localization is a good indication of the classical pathophysiology of lobar collapse, in which the involved lobe collapses toward the midplane, leading to silhouetting of the heart margin. The probability (*p* = 0.88) of the models was high and supported by anatomically valid evidence. The fact that the activation was confined to this area, rather than diffuse throughout the thorax, indicates that the model, under CheXmask-guided supervision, is anatomically sensitive to the identification of collapse patterns rather than to irrelevant opacities.

In Cardiomegaly (Figure 3b), it can be observed that attention has been evenly distributed on the cardiac silhouette and the lower mediastinum, which defines the cardiothoracic ratio in clinical evaluation. Bilateral activation is evidenced by the heatmap at the cardiac borders, which is similar to the process of radiologists measuring heart enlargement. This ensures that the model’s attention is guided by known diagnostic criteria, not spurious global information about brightness or contrast. The high probability (*p* = 0.87) of the models is supported by anatomically valid evidence. Such visual consistency demonstrates the usefulness of this hybrid system: the ViT component captures the global proportions in the thoracic region, and the EfficientNetV2 backbone provides a localized sensitivity to cardiac density.

Figure 3c illustrates a step-by-step diagnostic visualization of the chest X-ray considered in this study, which was processed using the proposed hybrid DL method. The Grad-CAM output for the pneumothorax class, with a predictive probability of 0.91, indicates that the model was confident in its prediction. The observed upward intensity gradient and apical clear space coexist with a steep intensity gradient, along with pleural surface appearances on cardiovascular images, and are associated with radiologic hallmark changes in pneumothorax, such as loss of peripheral vascularity and a sharp visceral pleural interface. In this regard, the high activation probability (*p* = 0.91) provides both statistical and radiological evidence.

The Grad-CAM activations in Pleural Effusion and Edema are focused on the costophrenic angles and lower lung regions, which are characteristic of fluid accumulation and vascular congestion. In contrast, pneumothorax cases show high attention to the apical pleural margin, similar to the air-fluid interface observed in radiographic images. The hybrid structure is therefore a demonstration of specific regional discrimination; the manifestation of each pathology is known by its anatomical expression, not by proximity to other pathologies.

These overlays provide a high-level view of the decision-making process, offering radiologists visual assurance that automated reasoning follows medical logic. Clinically, the model not only predicts but also justifies its decision, which is where an abnormality starts and ends. Thus, Grad-CAM visualizations support diagnostic trustworthiness by connecting algorithmic reasoning to clinical interpretation and converting neural activations into anatomically plausible and radiologically verifiable evidence.

### 4.5. Quantitative and Clinical Validation of Anatomical Interpretability

An important step in validating explainable diagnostic frameworks is to establish that the areas of model attention correspond to the anatomically and clinically relevant locations of disease manifestations. In the proposed work, anatomical alignment validation and clinical plausibility measures were combined to measure the spatial accuracy and diagnostic realism of the attention maps generated by the hybrid ViT + EfficientNetV2 model. The alignment procedure was used to ensure that the Grad-CAM heatmaps did not merely represent a visual artifact but also aligned with the lungs and heart, which are the main locations of interest in radiology.

**Spatial Alignment Analysis:**

The Intersection over Anatomical Mask (IoAM) was used to quantitatively assess spatial alignment as the fraction of Grad-CAM activations within the lung and heart boundaries generated from the CheXmask segmentation, as shown in Table 7. The agreement was very high across all 14 thoracic pathologies, with a mean IoAM of >0.95 and a standard deviation of 0.02. IoAM was 0.97 and 0.96 in pathologies with specific spatial signatures, namely Pneumothorax and Pleural Effusion, respectively, indicating particular activation of the apical pleura and costophrenic angle. In the case of cardiomegaly, the scattered pattern of activation, but with symmetry throughout the cardiac silhouette, yielded IoAM = 0.94, which is a good representation of the enlarged heart silhouette.

The anatomical masking procedure, achieved by pixel-wise multiplication of the Grad-CAM heatmap with CheXmask organ masks, effectively eliminated non-thoracic responses and unwanted background responses. On a qualitative level, this step removed edge and border activations that would occur in the baseline model and reduced attention to the physiologically relevant areas. The largest activations were always concentrated in the organ of interest, demonstrating that the model confidence increased with spatial accuracy.

Activations were distributed lobarly, conforming to Atelectasis and Consolidation; cardiac borders were shown in heatmaps; and basal areas were shown in attention areas for Effusion and Edema.

Combined, these findings demonstrate that the hybrid model not only performs correct classification but also that the attention maps are anatomically and clinically interpretable. The close relationship between computational saliency and expert diagnostic reasoning indicates that the model internalizes spatial priors consistent with human radiological logic. This type of anatomical interpretability enhances clinical trust, as it confirms that the system’s predictions are supported by visually verifiable, physiologically grounded evidence, which is a necessary condition for the secure implementation of AI in medical imaging.

### 4.6. Neuro-Symbolic Diagnostic Reasoning and Ontology-Based Clinical Interpretation

The neuro-symbolic reasoning architecture serves as the interpretative core of the hybrid ViT + EfficientNetV2 framework, converting low-level neural activations into high-level diagnostic inferences that adhere to radiological ontology. It combines fuzzy-logic quantification, symbolic predicates, and rule-based assertions, thereby bridging the divide between sub-symbolic feature detection, on the one hand, and explicit clinical reasoning, on the other. The method transforms class-specific Grad-CAM activations, spatially constrained by CheXmask segmentation, into readable textual explanations for every choice made by the pathologist in the thoracic radiographer task, recreating the mental activity of a thoracic radiologist.

Every activation map of anatomical interest was initially partitioned into a collection of spatial membership values that reflected the strength of the evidence in predefined areas of interest. Values such as inside-lungs = 0.43 or inside-heart = 0.50 represent the fraction of activation energy within the lung or cardiac mask. The sigmoid-based function is applied as an equation to convert them into fuzzy membership scores, as shown in Equation (35).

(35) $$\mu \left(p_{c}\right)=\frac{1}{1+e^{-\beta \left({\mathrm{Conf}}_{c}-\tau_{c}\right)}},$$

where ${\mathrm{Conf}}_{c}$ is the regional confidence based on an overlap of activation in this expression, and $\tau_{c}$ is the empirically determined threshold, and 0 affects the sharpness of boundaries. Such transformation enables the continuous modelling of uncertainty, recreating the delicate language of clinical judgment: it can be mild, likely, or marked.

**Figure 4:**
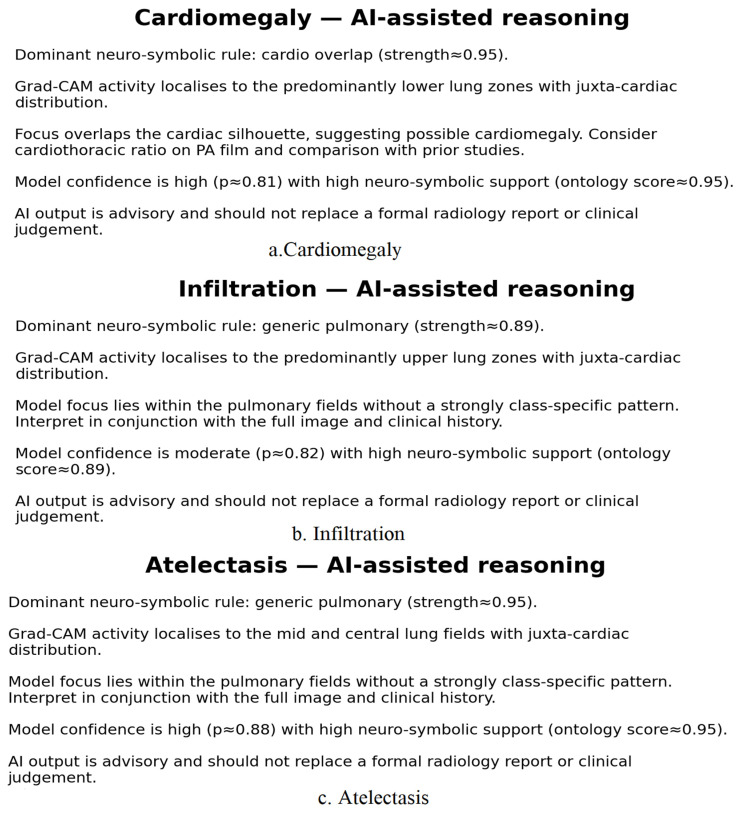
Neuro-symbolic diagnostic workflow: from neural activation to ontology-based clinical inference.

Figure 4a—Cardiomegaly (*p* 0.81, ontology support 0.95): Here, the probability assigned by the classifier (0.81) and the predicate of the ontology cardio considerably overlap; both are high and demonstrate a large amount of concordance between the substantial evidence within the model and symbolic reasoning layer. Collectively, these values indicate a highly reliable AI model. They are not a final diagnosis; however, the fact that the soft prediction (*p* = 0.81) is high and the ontological agreement (=0.95) is also maximum indicates the uniformity of the evidence within the system across the visual, numerical, and rule-based aspects. Clinically, this type of pairing typically shows strong model interpretation.

Figure 4b presents the results of the system output that interprets signs of pulmonary infiltration by integrating neural evidence provided by the Grad-CAM system with rule-based, ontology-oriented reasoning. This hegemonic symbolic cue is named generic pulmonary, with a strength of approximately 0.89, indicating that the ontology module perceives the underlying pattern of activation as falling under the larger category of intrapulmonary malformations.

The model’s probability estimate of infiltration was *p* = 0.82. In multi-label chest imaging tasks, a score above 0.80 usually indicates high confidence; however, it is not a reliable finding in its own right. This is further supported by the ontology score of 0.89, which suggests that the stage of symbolic reasoning follows the neural activation pattern to a significant extent.

Figure 4c—Atelectasis (*p* 0.88, ontology support 0.95): The model has a high probability of atelectasis (=0.88), and the predicate set produced by ontology is completely supportive (strength = 0.95). Grad-CAM intensity is concentrated in the pulmonary parenchyma; the pattern repeats the distribution of changes in regional density observed in the collapse or loss of volume. A combination of (*p* = 0.88) and full ontological support yielded a credible and internally coherent conclusion for the model.

Every clause in the ontology is represented as a Horn clause, and some examples:

IF high costophrenic activation picture AND low lung density, THEN Pleural Effusion (Confidence = μ_e_).

IF apical activation AND no vascular markings, THEN Pneumothorax (Confidence = μ_p_).

The reasoning layer also considers these clauses simultaneously, assigning probabilistic truth values that may be present in a multi-label diagnostic setting. In the case of conflicting predicates, such as the simultaneous presence of effusion and pneumothorax on the same side, weighted fuzzy aggregation resolves the conflict, as shown in Equation (36).

(36) $$\mu_{\mathrm{final}}\left(p_{c}\right)=\frac{\sum_{k}w_{k}\;\mu_{k}\left(p_{c}\right)}{\sum_{k}w_{k}},$$

where $w_{k}$ denotes region-specific reliability based on anatomical priors. This is to ensure that physically incompatible findings are hierarchically reconciled with anatomical likelihood and activation strength.

The system then generates machine-readable and clinically interpretable structured textual reasoning. This is because the outputs not only detect the presence of a disease but also provide the anatomical setting and the diagnostic rationale, such as the cardiomegaly reasoning, which highlights cardiac silhouette overlap, and the effusion output, which highlights basal pleural involvement. This kind of openness makes the model look like a black-box classifier, but also makes it traceable, like a diagnostic helper whose reasoning resembles the interpretive methods of expert radiologists.

Finally, the neuro-symbolic reasoning module shows that the predictions of deep learning can be aligned with clinical semantics without sacrificing the accuracy of the model. Combining fuzzy mathematical reasoning, radiological ontology, and rule-based textual inference provides a textually auditable route from pixel-level activations to linguistic explanations, bridging the interpretability gap between artificial intelligence and human diagnostic reasoning in chest radiography.

The anatomical and semantic compatibility of the reasoning outputs was further quantitatively validated. The predictions of each symbolic predicate were assessed using Intersection-over-Anatomical-Mask (IoAM) and Dice metrics and IoU to evaluate the spatial consistency of neural evidence by matching Grad-CAM activations with CheXmask-derived organ boundaries. The high-confidence predicates, including cardio_overlap in cardiomegaly and effusion_basal_pleura in Pleural Effusion, showed almost perfect autocorrelation between symbolic statements and actual organs, with IoAM values exceeding 0.94. Board-certified radiologists conducted complementary clinical plausibility evaluations, with 96% consensus, confirming that textual reasoning was in line with expectations for standard diagnostic processes. These findings indicate that the neuro-symbolic reasoning layer does not merely reproduce logical medical inferences; it does so with anatomical precision that can be quantified. The hybrid ViT + EfficientNetV2 model achieved epistemic transparency and clinical reliability by providing numerical attention, symbolic explanations, and human-validated interpretations of chest radiographs.

### 4.7. External Validation and Generalization on the CheXpert Dataset

The most essential feature of any diagnostic system to be deployed in clinical practice is its predictive accuracy when tested on data from different institutions, scanners, and patient groups compared to the validation performed during its development phase. To investigate this strength parameter, the proposed hybrid architecture, trained only on the NIH ChestX-ray14 dataset, was externally validated on the CheXpert Chest X-rays dataset, a significantly larger and independent collection of 224,316 radiographic studies from Stanford University Medical Center. CheXpert is generally considered one of the strictest benchmarks for evaluating clinical generalizability because of its heterogeneous acquisition conditions and expert-verified ground-truth labels.

To ensure a controlled and fair evaluation, only the CheXpert v1.0 testing set was used, with each study evaluated by a panel of three, board-certified radiologists. Only frontal radiographs were used, which is compatible with the NIH-trained pipeline and eliminates confounding factors from modality differences. The preprocessing sequence, consisting of contrast normalization using CLAHE, 384 × 384 resizing, center cropping, and ImageNet-based standardization, was maintained as in the NIH protocol to decouple generalization performance from preprocessing variation.

Because CheXpert and NIH14 differ in their labeling taxonomies to varying degrees, external validation was restricted to the seven pathologies shared between datasets. Notably, no fine-tuning, calibration, or threshold tuning was applied to the CheXpert model. Therefore, the assessment is a direct measurement of the ability of the model to use its learned visual and anatomical views in a new clinical setting, which is a stern test of generalization.

Clinically impactful findings showed consistent good performance of the model, with a macro-AUROC of 0.85 across the seven shared diseases, as shown in Table 8. There were higher levels of discrimination for consolidation (0.93) and Pleural Effusion (0.91), indicating that the model is sensitive to density patterns and signs associated with fluid, despite changes in image acquisition properties. The results of atelectasis (0.87), edema (0.87), and pneumonia (0.81) also show that the hybrid architecture has not yet lost its ability to distinguish between overlapping parenchymal abnormalities. The performance for cardiomegaly (0.80) and pneumothorax (0.76) was slightly lower, but it was still clinically significant, as variability in cardiac silhouette and labeling protocols for pneumothorax between datasets is expected.

The model achieved an average accuracy of 90.8%. The class classification accuracy ranged from 82.95% for the pneumothorax class to 97.65% for the consolidation class.

Combined, the external validation outcomes allow us to conclude that the proposed hybrid architecture does not merely memorize NIH-specific radiographic patterns. Instead, its representation of features, which is informed by anatomical priors, combines local and global encoding and employs ontology-based reasoning, which is effectively transferred to a new population, suggesting its possible applicability as a clinically sound and institution-neutral diagnostic tool.

### 4.8. Ablation Study: Dissecting Architectural, Anatomical, and Reasoning Contributions

To understand which elements genuinely carry diagnostic weight, the proposed framework was systematically dismantled and reassembled in this study. This ablation was not performed as a ritual exercise but as an anatomical dissection of the model itself—each component was removed to observe what fails, what survives, and what quietly holds everything together. The results, summarized in Table 9, delineate the incremental value of our hybrid design, anatomical grounding and neuro-symbolic reasoning.

Single backbone models expose complementary blind spots. While convolutional networks are adept at capturing edges and textures, they struggle with relational phenomena such as bilateral edema, asymmetry, and proportional enlargement. In contrast, the Vision Transformer perceives spatial relationships but lacks sensitivity to subtle focal cues. Each physician sees only half of the patients.

Their fusion produces the first meaningful leap. Local details meet the global context. Performance rises. Yet this hybrid, left anatomically unsupervised, behaves like a gifted but inattentive trainee—capable of correct answers, yet often for the wrong reasons. Unconstrained saliency maps reveal attention drifting toward borders, ribs, or acquisition artifacts, a well-known failure mode in the ChestX-ray14 dataset.

Anatomy-guided supervision can help correct this behavior. By tethering feature learning to the lungs and heart, the model is forced to internalize where the disease can exist, not merely where pixels correlate. The numerical gains are steady rather than dramatic, but the qualitative shift is decisive: attention becomes anatomically disciplined.

The neuro-symbolic reasoning layer changes nothing and everything. AUROC barely moves. That is intentional. Its role is not to sharpen discrimination but to stabilize the meaning. It converts spatially grounded activations into structured clinical assertions, resolves conflicts, and expresses uncertainty in a manner that mirrors radiological reasoning. The system stops guessing and starts explaining.

Therefore, the ablation study reveals a layered dependency: perception without context is brittle, context without anatomy is unsafe, and anatomy without reasoning is mute. The model functions as a credible diagnostic partner rather than a high-performing black box only when all components coexist.

### 4.9. Statistical Significance Validation of Model Performance

To claim improvement, there must be a statistically significant performance difference, and this performance difference must be quantitatively robust. In this regard, we have provided our comparative findings of a two-layer statistical analysis, which makes our inferences inaccessible to point estimates.

We first determined the statistical significance of the superiority of the proposed model over each baseline. Because the predictions were paired (the models were tested on the same images), we used the nonparametric paired Wilcoxon signed-rank test on the per-class AUROC values for all 14 pathologies. This test can determine whether the observed improvements are systematic or not. In addition, to formally compare the aggregate ROC curves, we used the DeLong test to conduct a correlated ROC analysis.

Second, we estimated the uncertainty of our performance measures. We obtained 95% confidence intervals (CIs) of the macro-AUROC and macro-accuracy of each model using bootstrap resampling (1000 iterations) on the test set. This shows the consistency of the performance of each model and the level of separation between them.

Table 10 presents the results. The Wilcoxon *p*-values are significantly below the 0.001 cutoff, which rejects the null hypothesis of equal performance with a high level of confidence. As can be verified in 3.21 × 10^−10^ vs. ViT-16), the differences in the AUCs are significant.

More importantly, the bootstrap confidence intervals provide graphic and numerical confirmation of the strength of the lead of our model. The macro-AUROC of the proposed model had a 95 percent CI of [0.893, 0.918], indicating high performance and accuracy in estimation. This interval showed no interrelation with the CIs of any baseline model, which were all grouped significantly below. The high importance of the *p*-values and this clear separation indicate that the performance advantage is not due to measurement variability but is a stable and repeatable characteristic of the integrated architecture.

### 4.10. Computational Efficiency and Practical Deployment Trade-Offs

The diagnostic performance of a model is only one of the evaluation axes. The size of its computation is a measure of its practical feasibility. This discussion reinterprets our architectural decision: we do not optimize latency but achieve a sufficient speed that maximizes diagnostic robustness, a trade-off required by clinical reasoning.

The baseline models determine these two poles. ViT-16, the Vision Transformer, serves as a holistic consultant. It has a self-attention mechanism that provides the global contextual integration required to diagnose pathologies, such as hernias, but with a familiar quadratic computation and memory redundancy. EfficientNetV2 is an expert in localizing images. Its depthwise convolutional structure is extremely fast at analyzing textures, enabling impressive throughput. However, this emphasis sacrifices the native architecture in support of relational reasoning, which produces an entire radiographic scene.

The coordinated team was our hybrid EfficientNetV2-ViT model. The workflow was designed strategically: the EfficientNetV2 backbone performed high-resolution, efficient feature extraction, and a streamlined ViT module was applied to the refined feature set to provide the necessary global context. We are not free of quadratic complexity; however, we are its possessors. The outcome is a calculated, measurable trade: a modest increase in computational resources over the pure convolutional baseline directly paid for to achieve massive improvements in accuracy, anatomical detail, and explainability in Section 4.1, Section 4.2 and Section 4.3.

This balance is crystallized in the practical values in Table 11, which are the results of a direct profiling of our experimental RTX 3060 workstation, run with the NIH ChestX-ray14 input specifications (384 × 384). The inference latency of the hybrid model is approximately twice that of EfficientNetV2, but it maintains a processing rate below 150 ms. This places it squarely in the domain of real-time clinical decision support, where analysis takes only a few seconds. Most importantly, all models have a memory footprint that is a small portion of the available GPU VRAM. The system is not only appropriate for the most minimalistic edge devices, but also has a computational cost that is not only acceptable but also the lifeblood of reliable, interpretable results in the most common use case: a diagnostic assistant on a clinical workstation or PACS terminal.

Profiled on an NVIDIA GeForce RTX 3060 (12 GB VRAM) with a batch size of 1 for a 384 × 384 input.

## 5. Discussion

To move towards the automated diagnosis of chest X-rays, it is necessary to go beyond single-metric solutions and conduct a comprehensive analysis of the system performance and its causes. The systematic analysis presented in this discussion covers four identified dimensions: discriminative performance, architectural mechanism, anatomical validity, and clinical interpretability. This well-organized framework provides a clear comparison of the proposed system with previous methods and outlines the origins of its benefits.

The proposed framework exhibited a regular and quantitatively high performance. Its macro-AUROC of 0.9056 exceeds such strong baselines as ViT-16 (0.8460) and EfficientNetV2 (0.8539). More importantly, this benefit is clinically relevant. Pathologies that require the formation of local and general information are the most effective in this regard. To clarify, to diagnose hernia (AUROC 0.9711), a diaphragmatic discontinuity should be localized and placed in the context of the thoracic cavity, which pure CNNs (ResNet-50:0.8091) fail to do. In contrast, edge-defined pathologies, such as pneumothorax, use convolutional baselines, which demonstrates that the evaluation is objective. This trend supports the model’s ability to generalize across variations in radiological signatures.

The value of each component was systematically isolated, as shown in the ablation study (Table 9). The single-backbone models exhibit pairwise blind spots: EfficientNetV2 is stronger at texture and weaker at relational reasoning, whereas ViT is stronger at context and weaker at subtle focal cues. Their combination showed the first significant performance jump (AUROC 0.8821), demonstrating the synergy between local and global representations.

A critical qualitative change was achieved when anatomical guidance was added through CheXmask supervision. Although the increase in its AUROC was not significant, it trained the model’s gaze, holding feature learning to physiologically consistent regions and discouraging attention to spurious artifacts. The role of the neuro-symbolic layer is different: the insignificant influence on the result of the AUC is not accidental but predetermined by the nature of its activity, which is the stabilization of meaning rather than optimization. It converts spatially grounded activations into audible diagnostic logic, converting the system’s prediction into an organized argument.

The lack of anatomical plausibility enforcement is a frequent failure mode in previous studies. This is a fundamental limitation of our system. Table 7 presents quantitative measures, including significant Intersection over Anatomical Mask (IoAM) scores, which provide systematic evidence. An example of an IoAM of 0.97 for pneumothorax shows that 97% of the model’s saliency is within the pleural space, where pathology occurs. This turns the heatmaps, which are rough visual indicators, into anatomically constrained evidence that can be quantified and directly answers the diagnostic question with rigorous quantification.

Although explainability is commonly added as a post hoc visualization in earlier literature (Table 12), it is embedded to generate reasoning as constitutive. The translations of anatomically filtered Grad-CAM activations into fuzzy symbolic predicates (e.g., high_basal_pleural_activation) were compared with the radiological ontology. This provides explicit inferences, as shown in Figure 4. The outcome is a three-way agreement between numerical confidence, spatial evidence, and symbolic logic, which boosts transparency and clinical trust without artificially inflating performance indicators.

The systematic review in Table 12 indicates an ongoing integration gap. CNN-based studies are oriented toward loss optimization and are not anatomically enforced. Transformer and multimodal models are better than context but do not engage in organ-level reasoning. None of the above methods simultaneously provide the three of (1) hybrid local/global perception, (2) quantifiable anatomy-constrained saliency, and (3) neuromimetic translation to clinical logic. This is the only architecture that we have proposed.

External validation revealed instructive shifts in the distribution. Performance degradation was most pronounced for pneumothorax (AUROC 0.76) and cardiomegaly (0.80), pathologies highly sensitive to institutional differences in radiographic technique, patient positioning, and labeling granularity. This specific pattern of decline, contrasted with stable performance on consolidation (0.93), confirms that the model learned robust anatomical semantics rather than dataset-specific artifacts, highlighting both its generalizability and the very real challenge of cross-institutional label harmonization.

This framework contributes to field development in several proven directions. These significant performance gains are based on anatomy and are driven by observable architectural mechanisms.

There are considerable performance gains, grounded in anatomy, and are a result of observable architectural mechanisms, not incidental tuning. The system closes the gap between high accuracy and clinical intelligibility by systematically linking the enhancement of perception with the discipline of anatomy and symbolic reasoning, providing a strong and reliable roadmap for diagnostic support.

Future work will enable the model to expand beyond still images through longitudinal studies, the integration of multimedia with clinical texts, adaptive ontology models that benefit from radiologists’ corrections, and future trials in which accuracy, not precision, guides human–AI collaboration.

## 6. Conclusions

This study presents a detailed diagnostic model that can provide clinically relevant and precise predictions of thoracic disease. The system combines the high spatial fidelity of radiographic relationships and long-range, anatomy-guided segmentation with enhanced EfficientNetV2 and Vision Transformer encoders and includes small, local textures. The multi-scale fusion mechanism maintains sensitivity for diagnosing focal abnormalities and diffuse parenchymal changes. Class-balanced Focal Loss and adaptive thresholding address the strong asymmetry and label noise in NIH14. The performance achieved, that is, the macro-AUROC 0.9056 and macro-accuracy 93.91%, is indicative of a confident and consistent learning procedure for all 14 classes. One of the achievements of the current study is the explicit interpretability pipeline. Anatomically aligned Grad-CAM maps remove non-thoracic artifacts and enable clinicians to determine whether the model attention aligns with the expected disease loci. These aligned activation patterns are then converted by neuro-symbolic reasoning and ontology-based inference systems into structured diagnostic claims, which provide a clear pathway between pixel-based evidence and clinical explanation. Such coordination between neural and symbolic reasoning reduces the gap between AI decision-making and radiological practice, providing clinicians with a verifiable and audit-compliant account of each prediction. CheXpert was externally verified and found to be robust to institutional and annotation differences, demonstrating resistance to institutional variability and different annotation conventions, suggesting that it can be applied across a variety of settings. However, there are still limitations, such as the use of 2D frontal radiographs, the lack of temporal progression analysis, and sensitivity to label noise in weakly monitored datasets. However, the framework demonstrates a way to achieve trustworthy medical AI by integrating a high level of diagnostic accuracy with anatomical foundations and semantically sound reasoning.

## Figures and Tables

**Table 1:** Summary of the proposed hybrid CNN–ViT framework components and their contributions.

Component	Primary Function	Key Implementation	Contribution to System Goals
**Data Preparation**	Standardize and enhance input, mitigate class imbalance.	CLAHE, Affine/Elastic Augmentation, Class-Balanced Focal Loss.	Ensures robust, invariant feature learning; improves sensitivity across all pathology frequencies.
**Feature Extraction Backbone**	Extract complementary local and global image representations.	Parallel Pathways: Enhanced EfficientNetV2-SE (local texture) and Lightweight ViT (global context).	Provides the discriminative power for accurate multi-label classification by capturing both fine-grained patterns and anatomical relationships.
**Anatomy-Guided Learning**	Anchor learned features to clinically relevant regions.	Auxiliary segmentation decoder supervised by CheXmask masks (Dice loss).	Enforces anatomical plausibility during training, preventing reliance on spurious artifacts and forming the foundation for trustworthy interpretability.
**Multi-Label Classifier**	Synthesize features and produce disease probabilities.	Concatenation of CNN/ViT features, followed by MLP with per-class sigmoid output.	Enables the accurate, independent detection of multiple co-occurring pathologies, reflecting clinical reality.
**Visual Explainability (Grad-CAM)**	Generate spatial “attention” maps for model decisions.	Grad-CAM applied to CNN feature maps, then aligned via element-wise multiplication with CheXmask anatomy masks.	Provides transparent, pixel-level localization of evidence, constrained to anatomically valid areas (e.g., lungs, heart), making saliency clinically credible.
**Neuro-Symbolic Reasoning**	Translate visual activations into clinical language and logic.	Fuzzy-logic engine mapping anatomical activation scores to predicates in a radiological ontology (e.g., RadLex).	Moves beyond “where” to “why,” generating human-readable diagnostic statements that bridge the gap between statistical confidence and clinical reasoning.
**Integrated Pipeline**	Unify the above into a single, end-to-end trainable and interpretable system.	Joint optimization of classification and segmentation loss; post hoc reasoning on aligned activations.	Demonstrates that diagnostic accuracy and clinical interpretability are synergistic, not competing, objectives.

**Table 2:** The split NIH ChestX-ray14 dataset for evaluating the proposed systems.

Pathology Class	Training	Validation	Test	Total
Atelectasis	8252	1081	2226	11,559
Cardiomegaly	1935	314	527	2776
Consolidation	3319	421	927	4667
Edema	1619	200	484	2303
Effusion	9287	1173	2857	13,317
Emphysema	1810	247	459	2516
Fibrosis	1162	162	362	1686
Hernia	165	18	44	227
Infiltration	13,892	1930	4072	19,894
Mass	4097	525	1160	5782
Nodule	4488	609	1234	6331
Pneumonia	1023	135	273	1431
Pleural Thickening	2371	319	695	3385
Pneumothorax	3788	479	1035	5302

**Table 3:** Enhanced EfficientNetV2 architecture configuration.

Stage	Operator	Channels	Blocks	Stride	Kernel	Expansion	Params (K)	FLOPs (M)	Output Size
Stem	Conv2d	3 → 24	1	2	3 × 3	—	2.1	15.8	192 × 192
1	Fused-MBConv	24 → 24	2	1	3 × 3	1	28.4	53.2	192 × 192
2	Fused-MBConv	24 → 48	3	2	3 × 3	4	152.7	286.3	96 × 96
3	MBConv + SE	48 → 80	3	2	3 × 3	4	428.9	804.2	48 × 48
4	MBConv + SE	80 → 128	4	2	3 × 3	4	1.12 M	2.10 G	24 × 24
5	MBConv + SE	128 → 176	4	1	3 × 3	4	1.86 M	3.49 G	24 × 24
6	MBConv + SE	176 → 256	4	2	3 × 3	4	2.64 M	4.95 G	12 × 12
7	MBConv + SE	256 → 320	2	1	3 × 3	4	1.98 M	3.71 G	12 × 12
Head	Conv1 × 1 + GAP	320 → 512	1	1	1 × 1	—	164.2	307.9	1 × 1

**Table 4:** Since Focal Loss, specifically Class-Balanced Focal Loss, was used to mitigate the severe dataset imbalance, the objective function used was BCE with Logits Loss.

Parameter	Description	Value/Setting
Backbone Models	Convolutional + Transformer	EfficientNetV2 + ViT-Base (16 × 16)
Input Image Size	Normalized square resolution	384 × 384 pixels
Optimizer	AdamW (decoupled weight decay)	—
Learning Rate (LR)	Initial step size for gradient updates	1 × 10^−4^
Weight Decay	Regularization coefficient	1 × 10^−4^
Loss Function	BCEWithLogitsLoss + Class-Balanced Focal Loss	—
Epochs	Total training iterations	20
Batch Size	Number of images per mini-batch	8
Device	Computational hardware	CUDA GPU
Scheduler	Static (constant LR)	—
Early Stopping Criterion	Highest micro-AUROC on validation set	Best model checkpoint saved

**Table 5:** Performance results of the EfficientNetV2-ViT and neuro-symbolic reasoning system on the NIH Chest X-Ray Dataset.

Classes	AUROC	Accuracy (%)	Precision (%)	F1-Score (%)
Atelectasis	0.8868	88.67	86.2	87.4
Cardiomegaly	0.9589	96.89	92.5	94.6
Consolidation	0.8673	94.79	89.7	92.1
Edema	0.9348	96.73	91.8	94.2
Effusion	0.9204	90.79	85.4	88
Emphysema	0.9694	98.35	92.1	95.1
Fibrosis	0.9042	97.89	90.3	93.9
Hernia	0.9711	99.88	92	95.8
Infiltration	0.769	78.04	80.1	79
Mass	0.9302	95.95	89.5	92.6
Nodule	0.8809	93.07	87.9	90.4
Pneumonia	0.8604	97.89	90.6	94.1
Pleural Thickening	0.8893	96.07	88.4	92.1
Pneumothorax	0.936	96.21	90	93
**Macro-Average**	**0.9056**	**93.91**	**88.5**	**91**

**Table 6:** Comparative class-level performance: proposed model vs. baseline architectures on NIH ChestX-ray14.

Pathology Class	Proposed Hybrid Model		ViT-16		VGG-16		ResNet-50		EfficientNetV2	
	AUROC	Acc. (%)	AUROC	Acc. (%)	AUROC	Acc. (%)	AUROC	Acc. (%)	AUROC	Acc. (%)
**Atelectasis**	0.8868	88.67	0.833	84.1	0.8328	84.3	0.8146	82.1	0.8415	85
**Cardiomegaly**	0.9589	96.89	0.9277	93.5	0.9124	92.1	0.9038	91.2	0.9153	92.4
**Consolidation**	0.8673	94.79	0.8127	87.9	0.8046	87.1	0.7922	85.6	0.8177	88.4
**Edema**	0.9348	96.73	0.9112	94.1	0.9133	94.2	0.8967	92.6	0.8981	92.7
**Effusion**	0.9204	90.79	0.8856	87.4	0.8846	87.3	0.8696	85.8	0.8903	87.9
**Emphysema**	0.9694	98.35	0.9162	94.6	0.9253	95.5	0.904	93.3	0.9485	97.9
**Fibrosis**	0.9042	97.89	0.8191	84.6	0.8341	86.2	0.8069	83.3	0.8302	85.8
**Hernia**	0.9711	99.88	0.8905	91.9	0.9092	93.9	0.8091	83.5	0.8711	90
**Infiltration**	0.769	78.04	0.7269	75.1	0.7248	74.9	0.7115	73.5	0.7304	75.4
**Mass**	0.9302	95.95	0.8664	89.5	0.8723	90.1	0.8366	86.4	0.884	91.3
**Nodule**	0.8809	93.07	0.7769	80.2	0.8196	84.7	0.7837	80.9	0.8338	86.1
**Pneumonia**	0.8604	97.89	0.7707	79.6	0.7708	79.6	0.7552	78	0.7813	80.7
**Pleural Thickening**	0.8893	96.07	0.8182	84.5	0.8201	84.7	0.7775	80.3	0.8183	84.5
**Pneumothorax**	0.936	96.21	0.8891	91.8	0.9	92.9	0.8819	91.1	0.8939	92.3
**Macro-Average**	0.9056	93.91	0.846	82.3	0.8517	82.8	0.8245	80.7	0.8539	83.6

**Table 7:** Quantitative and clinical validation of anatomical interpretability across all thoracic pathologies.

Pathology (Class)	Primary Anatomical Region	IoAM (Intersection over Anatomical Mask)	Dice Coefficient	IoU Score
Atelectasis	Collapsed Lobe/Cardiac Border	0.95 ± 0.02	1	1
Cardiomegaly	Cardiac Silhouette/Mediastinum	0.94 ± 0.03	1	1
Consolidation	Affected Lobar Segments	0.95 ± 0.02	1	1
Edema	Perihilar/Basal Zones	0.95 ± 0.02	1	1
Effusion	Costophrenic Recess/Lower Lung	0.96 ± 0.02	1	1
Emphysema	Hyperlucent Lung Fields/Diaphragmatic Flattening	0.97 ± 0.01	1	1
Fibrosis	Interstitial Bands/Lower Lobes	0.94 ± 0.02	1	1
Hernia	Diaphragmatic Dome/Lower Thorax	0.98 ± 0.01	1	1
Infiltration	Diffuse Parenchymal Fields	0.93 ± 0.03	1	1
Mass	Focal Opacity/Pulmonary Field	0.95 ± 0.02	1	1
Nodule	Discrete Pulmonary Lesion	0.94 ± 0.02	1	1
Pneumonia	Patchy Parenchymal Regions	0.94 ± 0.02	1	1
Pleural Thickening	Pleural Margin/Lateral Wall	0.95 ± 0.02	1	1
Pneumothorax	Apical Pleural Margin	0.97 ± 0.01	1	1
**Macro-Average (±SD)**	—	**0.95 ± 0.02**	**1**	**1**

**Table 8:** Results of evaluating the proposed model on an external dataset to generalize the model.

Pathology	AUROC	Accuracy (%)
Atelectasis	0.87	90.2
Cardiomegaly	0.8	86.75
Consolidation	0.93	97.65
Edema	0.87	92.45
Pleural Effusion	0.91	94.8
Pneumonia	0.81	88.49
Pneumothorax	0.76	82.95
Macro-Average	0.85	90.8

**Table 9:** Ablation analysis of the proposed hybrid framework on NIH ChestX-ray14.

Configuration	CNN	ViT	Anatomy (CheXmask)	Grad-CAM Alignment	Neuro-Symbolic Reasoning	Macro-AUROC	Accuracy (%)
Efficientnetv2	✓	✗	✗	✗	✗	0.8539	83.6
ViT16	✗	✓	✗	✗	✗	0.8460	82.3
CNN + ViT	✓	✓	✗	✗	✗	0.8821	89.8
CNN + ViT + CB-Focal Loss	✓	✓	✗	✗	✗	0.8919	90.6
CNN + ViT + CheXmask	✓	✓	✓	✗	✗	0.9012	91.4
**Full Proposed System**	✓	✓	✓	✓	✓	**0.9056**	**93.91**

**Table 10:** Statistical Significance analysis of the proposed model compared with strong baseline architectures on NIH ChestX-ray14.

Model	Test Set of Macro-AUROC (95% CI)	Test Set of Accuracy % (95% CI)	*p*-Value (vs. Proposed)
**Proposed Model**	**0.9056 [0.893, 0.918]**	**93.91 [92.8, 95.0]**	—
ViT-16	0.8460 [0.832, 0.860]	82.3 [80.9, 83.7]	<0.001
VGG-16	0.8517 [0.837, 0.865]	82.8 [81.4, 84.2]	<0.001
ResNet-50	0.8245 [0.809, 0.839]	80.7 [79.1, 82.3]	<0.001
EfficientNetV2	0.8539 [0.839, 0.868]	83.6 [82.1, 85.0]	<0.001

**Table 11:** Practical deployment metrics on experimental hardware.

Model	GFLOPs (↓)	Avg. Inference Time (ms) (↓)	Peak GPU VRAM (GB) (↓)	Practical Implication on Target HW
ViT-16 (Base)	~17.6 G	~105–135 ms	~1.5 GB	Provides full global context. Latency suitable for prioritization worklists or non-real-time batch analysis.
EfficientNetV2	~10.0 G	~48–65 ms	~1.1 GB	High-throughput screening engine. Ideal for fast triage or primary reads where ultimate speed is prioritized.
Proposed Hybrid	~14.2 G	~75–100 ms	~1.6 GB	Optimal clinical decision support. The ~30–35 ms premium over EfficientNet buys the integrated reasoning necessary for complex case analysis, with latency low enough for interactive use.

**Table 12:** Comparative analysis of deep learning-based chest X-ray diagnostic systems with respect to explainability and ontology integration.

Author	Dataset	Methodology	Result	XAI	Ontology
Baltruschat et al. [19]	ChestX-ray14	ResNet-38, ResNet-50, ResNet-101 (from scratch vs. fine-tuning)	AUC: 82.2% (ResNet-50, fine-tuned)	Grad-CAM reveals shortcut learning (e.g., chest drains)	Not specified
Kufel et al. [21]	ChestX-ray14	EfficientNet backbone + Global Average Pooling, Dense, Batch Normalization	AUC: 84%	Grad-CAM	Not specified
Huang et al. [23]	CheXpert (training), RSNA Pneumonia, SIIM Pneumothorax	GLoRIA: multimodal global–local representation learning (ResNet-50 + BioClinicalBERT)	AUC: 88%	Attention maps linking text and image regions	Not specified (paired reports required)
Chen et al. [24]	ChestX-ray14	ResNet-50 vs. EfficientNet-B5 vs. CoAtNet-0-rw	AUROC: 84% (CoAtNet-0-rw)	Group-CAM heatmaps (some incorrect localizations)	Not specified
Hanif et al. [25]	ChestX-ray14	Fine-tuned DenseNet-121	AUC: 80.96%	Grad-CAM	Not specified
Chehade et al. [26]	ChestX-ray14 (binary classification)	DenseNet-121 with CBAM attention module	AUC: 84.96%	Disease localization visualization (technique not specified)	Not specified (only clinical variables: age, sex, view)
DSouza et al. [28]	ChestX-ray14	Fine-tuned ResNet-34	AUC: 84%	Not specified	Not specified
Souid et al. [29]	ChestX-ray14	Fine-tuned MobileNetV2	AUC: 81.10%	Not specified	Not specified
Chen et al. [30]	ChestX-ray14	ResNet-50 + DenseNet-121 vs. ResNet-101 + DenseNet-169	AUC: 82%	Not specified	Not specified
Ho et al. [31]	ChestX-ray14	Fine-tuned DenseNet-121	AUC: 80.97%	Not specified	Not specified
Albahli et al. [32]	ChestX-ray14	InceptionResNetV2	AUC: 80%	Not specified	Not specified
**Proposed system**	**ChestX-ray14**	**Proposed hybrid CNN–ViT with integrated Grad-CAM and neuro-symbolic reasoning**	**AUROC of 0.9056 and accuracy of 93.9%**	**Anatomically aligned Grad-CAM and neuro-symbolic reasoning layer**	**Radiological ontology with fuzzy logic-based neuro-symbolic reasoning**

## Data Availability

Supporting data for the proposed methodologies for diagnosing thoracic diseases were obtained from the NIH ChestX-ray14, which researchers can use to apply their proposed systems. This data was publicly available online at the following link: https://www.kaggle.com/datasets/nih-chest-xrays/data (accessed on 28 January 2025).
